# Astrocytic CCAAT/Enhancer Binding Protein δ Regulates Neuronal Viability and Spatial Learning Ability via miR-135a

**DOI:** 10.1007/s12035-015-9359-z

**Published:** 2015-07-26

**Authors:** Yu-Yi Chu, Chiung-Yuan Ko, Wei-Jan Wang, Shao-Ming Wang, Po-Wu Gean, Yu-Min Kuo, Ju-Ming Wang

**Affiliations:** 1 Institute of Bioinformatics and Biosignal Transduction, College of Bioscience and Biotechnology, National Cheng Kung University, Tainan, 701 Taiwan; 2 Graduate Institute of Neural Regenerative Medicine, College of Medical Science and Technology, Taipei Medical University, Taipei, 110 Taiwan; 3 Institute of Basic Medical Sciences, National Cheng Kung University, Tainan, 701 Taiwan; 4 Department of Pharmacology, National Cheng Kung University, Tainan, 701 Taiwan; 5 Department of Cell Biology and Anatomy, National Cheng Kung University, Tainan, 701 Taiwan; 6 Infectious Disease and Signaling Research Center, National Cheng Kung University, Tainan, 701 Taiwan; 7 Center for Neurotrauma and Neuroregeneration, Taipei Medical University, Taipei, 110 Taiwan; 8 Institute of Medical Sciences, College of Medicine, Taipei Medical University, Taipei, 110 Taiwan

**Keywords:** miR-135a, Neuronal viability, Spatial learning ability, Neuroinflammation, Neuroprotective factor

## Abstract

The progression of Alzheimer’s disease (AD) has been associated with astrocytes-induced neuroinflammation. However, the detailed mechanism of astrocytes associated with learning impairments and neuronal loss in AD is poorly defined. Here, we provide novel evidences that astrocytic miR-135a is critical for neuronal viability and spatial learning ability in vivo. The *AppTg*/*Cebpd* ^−/−^ mice showed a spatial learning improvement compared with the APPswe/PS1/E9 bigenic (*App*Tg) mice. miR-135a was found to be a CCAAT/enhancer binding protein δ (CEBPD) responsive miRNA and can repress the transcription of thrombospondin 1 (THBS1) / Thbs1 (mouse) via its 3′-untranslated region (3′UTR). We used different experimental approaches to attenuate the expression of CEBPD/Cebpd (mouse) or miR-135a in astrocytes and found the following results: increase in THBS1/Thbs1 expression, decrease in neuronal apoptosis, and increase in growth of neurites. Importantly, injection of miR-135a antagonist (AM135a) into the brain of *AppTg* mice was found to prevent neuronal apoptosis and improved the spatial learning ability. Together, our findings demonstrate a critical function for the astrocytic CEBPD, and point to miR-135a antagonist as an attractive therapeutic target for the treatment of Alzheimer’s disease.

**Electronic supplementary material:**

The online version of this article (doi:10.1007/s12035-015-9359-z) contains supplementary material, which is available to authorized users.

## Introduction

Neuroinflammation is a prominent feature of Alzheimer’s disease (AD) and has been suggested to play a role in AD progression [1–3]. Current consensuses suggest that neuroinflammation can lead to neuronal dysfunction, death, and ultimately severe cognitive impairment of AD patient [4, 5]. Accumulated results found that the reactive astrocytes are involved in the induction of neuroinflammation [6–8]. Astrocytes are originally thought to be supportive glial cell components in the central nervous system (CNS), which provide energy metabolites to neurons and regulate neuron maturation [9, 10]. Recently, it has also been recognized that the secretory function of astrocytes can further regulate neuronal survival and neuronal repair after CNS injury [11]. Thrombospondin 1 (THBS1) is a multimeric extracellular matrix glycoprotein [12] secreted by astrocytes, which can function in neuronal dendritic growth, axonal sprouting, and synaptogenesis [13–15].

CCAAT/enhancer binding protein delta (CEBPD) is a pivotal transcription factor in brain inflammation and is a member of CCAAT/enhancer binding protein (C/EBP) family [16]. CEBPD is responsive to many proinflammatory cytokines involved in the pathogenesis of neurodegenerative diseases [17, 18, 1] such as interleukin-1β (IL-1β), interleukin-6 (IL-6), and tumor necrosis factor α (TNFα) [19, 1]. Interestingly, the expression of CEBPD was found to be increased in age-associated disorders including AD [20], atherosclerosis [21], type 2 diabetes [22], and RA [23]. We have previously tried to attenuate the expression of CEBPD and found that it can lead to better prognosis in mouse model of AD [24, 25]. The better AD prognosis has been attributed to attenuation in endothelial cell-mediated angiogenesis and macrophage-mediated phagocytosis of damaged neurons and increase in anti-apoptosis effect of astrocytes [26, 25, 27].

The role of CEBPD in the pathogenesis of AD has been investigated, but there are limited studies on the signal transduction pathway that CEBPD undertook in the pathogenesis of AD. Specifically, we were interested to investigate the downstream miRNA targets of astrocytic CEBPD and how it can lead to neuroinflammation. miRNAs are small non-coding RNA that regulate gene expression post-transcriptionally by base-pairing to mRNA [28]. There is evidence that imbalanced in the expression of miRNAs can lead to neurodegenerative diseases [29–31] and differential expression of miRNA in astrocytes and neurons after ischemic injury [32].

Glia-mediated neurotoxicity and neuroprotection have been suggested to be involved in the cognitive loss of AD patients. However, astrocytes participate in neuronal loss and consequent learning impairments in AD is poorly defined. Transcription factor CEBPD is significantly activated in astrocytes of AD patients and *App*Tg mice [20, 26]. In this study, the deficit of spatial learning ability in *AppTg* mice was ameliorated in *AppTg*/*Cebpd* ^−/−^ mice. Therefore, the investigation of CEBPD biology in astrocytes, especially in regulation of neurotrophic factors, could provide a new insight for understanding the communication of astrocytes and neuronal cells in AD pathogenesis. Our results suggested that astrocytic CEBPD activation results in the impairment of spatial learning ability through activating miR-135a to suppress neurotropic factor THBS1. Importantly, the miR-135a antagonist (AM135a) can effectively recuse the spatial learning ability of *AppTg* mice.

## Materials and Methods

### Materials

α-Tubulin antibody (T6199) was purchased from Sigma (St. Louis, MO, USA) and CEBPD antibody (SC-636) was purchased from Santa Cruz Biotechnology (Santa Cruz, CA, USA). NeuN antibody (MAB377) was purchased from Merck Millipore. The cleaved caspase 3 antibody (#9661) was purchased from Cell Signaling Technology (Danvers, MA, USA). The antibodies against THBS1 (GTX21823) and MAP2 (GTX11267) were purchased from GeneTex (Irvine, CA, USA). Secondary antibodies used in immunofluorescence analysis were goat anti-mouse/rabbit IgG (H + L) secondary antibody, Alexa Fluor® 488 conjugate (A-11001/A-11008) and goat anti-mouse/rabbit IgG (H + L) secondary antibody, Alexa Fluor® 568 conjugate (A-11031/A-11011) obtained from Invitrogen (Carlsbad, CA, USA). Secondary antibodies used in Western blot analysis were goat anti-mouse/rabbit IgG, peroxidase-conjugated (AP-124P/AP-132P) purchased from Merck Millipore. TRIzol RNA extraction reagent, Dulbecco’s modified Eagle’s medium (DMEM), and Opti-MEM medium were obtained from Invitrogen (Carlsbad, CA, USA). All oligonucleotides were synthesized by MDBio Inc. (Taipei, Taiwan). Fetal bovine serum (FBS) was purchased from HyClone Laboratories (Logan, UT, USA).

### Animals

The *Cebpd*-deficient mice were a gift from Dr. E. Sterneck. The *APPswe*/*PS1*/*E9 transgenic* (*App*Tg) *mice* were obtained from Jackson Laboratory (Bar Harbor, ME, USA, stock no. 004462). The *App*Tg mice were crossed with *Cebpd* ^−/−^ mice on the C57BL/6 genetic background. Female mice heterozygous for *App*Tg mice was intercrossed with *Cebpd* ^−/−^ homozygous mice; the offspring (*App*Tg^+/−^/*Cebpd* ^+/−^) were then bred to each other to produce the *App*Tg/*Cebpd* ^−/−^ mice in this study. The 12 month aged wild-type (*Cebpd* ^+/+^), *App*Tg, and *AppTg*/*Cebpd* ^−/−^ mice were used to perform the Morris water maze in this study. The *N* number of each group is 3–5.

### Cell Culture and Preparation of Primary Cells

U373MG (human glioblastoma-astrocytoma) and SH-SY5Y (human neuroblastoma) cells were cultured in DMEM or DMEM/F12 containing 10 % FBS, 100 μg/mL streptomycin, and 100 units/ml penicillin. For the preparation of primary mouse astrocytes and cortical neuron, the cortex were removed from P0 mouse brains and dissected in HEPES-buffered saline, carefully stripped of their meninges and digested with 0.25 % trypsin in Hanks’ Balanced Salt Solution (Invitrogen, Carlsbad, CA, USA) for 10 min at 37 °C. Trypsinization was stopped by adding an equal volume of culture medium. Cortical neurons were dissociated specifically by trituration in neurobasal plus B27 supplemented with 1 % l-glutamine and 1 % penicillin medium and passage through a 70-μm nylon strainer. The solution was pelleted (10 min, 200 g) and then resuspended in culture medium and brought to a single cell suspension by repeated pipetting followed by passage through a 70-μm nylon strainer. Later, astrocytes were seeded on plates coated with laminin (20 μg/mL, Invitrogen). Laminin-coating favors astroglial growth and inhibits microglial growth [33]. Cortical neurons were then plated on glass slides or plastic culture dishes coated with poly-l-lysine (Sigma).

### Immunofluorescence Analysis

The frozen mouse brain sections were treated with protein blocker/antibody diluents (Bio SB) for 1 h. In the same buffer solution, the sections were incubated overnight with primary antibodies at 4 °C. These primary antibodies included caspase 3 and MAP2. For the staining of cultured cells, primary cortical neuron cells were post-fixed in 4 % paraformaldehyde in phosphate-buffered saline (PBS) for 10 min, followed by 0.5 % Triton X-100 in PBS at room temperature for 10 min. The fixed neuron cells were further incubated with primary antibodies overnight against target proteins in 3 % bovine serum albumin at 4 °C. Pretreated slides of the tissue sections or neuron cells were washed with 0.05 % Tween-20 in PBS and then incubated with Alexa488- or 568-conjugated secondary antibodies for 1 h then washed again with 0.05 % Tween-20 in PBS. Next, the glass slides were counter-stained and mounted with ProLong Gold antifade reagent with 4′, 6-diamidino-2-phenylindole for immunofluorescence microscopy.

### The Preparation of Conditioned Medium

For the collection of CM, wild-type and *Cebpd*-deficient primary astrocytes were pretreated with or without 5 ng/mL IL-1β (Invitrogen, Carlsbad, CA, USA) for 6 h. Later, the IL-1β pretreated cells were washed by PBS and replaced in the serum-free medium. After additional 12 h, the supernatants were collected after centrifuge at 5000 g for conditioned media.

### Cell Survival Assays

For the neuronal cell survival assay, P0 wild-type and *Cebpd*-deficient primary cortical neurons were plated and maintained for 7 days. The experimental cells were grown in the conditioned medium as mentioned above for 48 h. Next, the media was removed and replaced with diluted 3-(4,5-cimethylthiazol-2-yl)-2,5-diphenyl tetrazolium bromide (MTT) reagent for 4 h. The samples were then measured spectrophotometrically at 595 nm using an enzyme-linked immunosorbent assay plate reader.

### Microarray Analysis

Total RNAs were isolated using the TRIzol RNA extraction reagent. Samples were validated with Agilent Human and Primate miRNA one array (Phalanx Biotech., Taipei, Taiwan), following the manufacturers’ protocols. All processes were performed by Phalanx Biotech Company (Taipei, Taiwan). Good quality signals were obtained by filtering for scores of *p* value <0.05 in all replicates, *M* value of >6 in all signals, and more than 1.5-fold change.

### RNA Isolation, Reverse Transcription, and Quantitative Real-Time PCR

Total RNAs were isolated using the TRIzol RNA extraction reagent and subjected to reverse transcription with SuperScriptTM III. Specific primers used for the RT-PCR analysis are as follows: for *GAPDH* (20 cycles and product size: 576 bp), 5′-CCATCACCATCTTCCAGGAG-3′ and 5′-CCTGCTTCACCACCTTCTTG-3′; for *THBS1* (28 cycles and product size: 376 bp), 5′-GCTCAGAGTGGATGTTATGG-3′ and 5′-GGGAATACTTCTCTGCAGAG-3′; for *Thbs1* (28 cycles and product size: 192 bp), 5′-CCAAAGCCTGCAAGAAAGAC-3′ and 5′-CCTGCTTGTTGCAAACTTGA-3′; for *CEBPD* (26 cycles and product size: 267 bp), 5′-AGCGCAACAACATCGCCGTG-3′ and 5′-GTCGGGTCTGAGGTATGGGTC-3′; and for *Cebpd* (26 cycles and product size: 429 bp), 5′-ATCGCTGCAGCTTCCTATGT-3′ and 5′-GGTTAAGCCCGCAAACATTA-3′ The PCR products were separated by electrophoresis in 1 % agarose gels and visualized with ethidium bromide staining. For qRT-PCR, the resultant cDNA was mixed with SYBR Premix ExTaq kit and appropriate primers, and quantitative PCR was performed with Thermocycler C1000 (Bio-Rad, Hercules, CA, USA). Forty cycles were set for qRT-PCR program and the abundances of interested gene expression were calculated following the formula (2 ^-ΔΔCt^) suggested in the instruction of qRT-PCR kit (Applied Biosystems). Specific primers used for the qRT-PCR analysis are as follows: for *GAPDH*, 5′-CCACCCAGAAGACTGTGGAT-3′ and 5′-AAGGTCATCCCTGAGCTGAA-3′; for *THBS1*, 5′-GACCTGCCACATTCAGGAGT-3′ and 5′-CTTTCTTGCAGGCTTTGGTC-3′; for *Thbs1*, 5′-CCAAAGCCTGCAAGAAAGAC-3′ and 5′-CCTGCTTGTTGCAAACTTGA-3′; for miR-135a reverse transcription (RT) primer, 5′-CTCAACTGGTGTCGTGGAGTCGGCAATCACTTGAGTCACATAG-3′; and for miR-135a forward, 5′-ACACTCCAGCTCAGTATGGCTTTTTATTCCTATGT-3′ and reverse, 5′-CTCAACTGGTGTCGTGGAGTCGGCAATTCAG-3′.

### TaqMan Reverse Transcription-PCR for miRNA Quantification

Total RNA was isolated using TRIzol according to the manufacturer’s protocol, reverse-transcribed using a TaqMan® microRNA reverse transcription kit, and subjected to real-time PCR using a TaqMan® microRNA assay kit (Applied Biosystems).

### Cloning and Mutagenesis

The 3′-UTRs of *THBS1* and *Thbs1* genes and 5′-flanking region of GLYCTK-AS1-001 gene (host gene of intronic miR-135a) were cloned from U373MG cells by using the DNeasy Tissue Kit (QIAGEN, Düsseldorf, Germany) and PCR. The following primers were used for PCR and cloning: GLYCTK-AS1-001 forward, 5′-KpnI-CGGGGTACCCCGAGCCCCCTGCAACCT-3′ and reverse primer 5′Xho1-CCGCTCGAGCGGACCGAGAGATAAAGCCTG-3′; THBS1-3′UTR forward, 5′XbalI-TCTAGATGTAGCTTGTGCAGATGT-3′ and reverse, 5′EcoRV-CTGAGATATCTATTCCAATGGCAATGAG-3′; Thbs1-3′UTR (1w) forward, 5′Xbal1-TCTAGAGCCAATCATAACCAGC-3′ and reverse, 5′EcoRV-GATATCGTATAGAAGTTCCACCTTTGT-3′. These PCR fragments were subcloned into pGL3-promoter or pGL3-basic vector for further reporter assay. Mutant reporter plasmids were constructed by the site-directed mutagenesis following the instructions of the QuikChang Site-directed Mutagenesis Kit (Stratagene, CA, USA) with the primers of THBS1-3′UTR mutant, 5′-AAATTGCAAAGAAAGATATCAGGTCTTCAATACTGT-3′, M1, 5′-CTCCTTGTAATGGATATCAGGAGTACTCTA-3′, or M2, 5′-GTTTGCTTTTGGGATATCCAAAGCGCCTAT-3′.

### DNA Vector Transfection and Reporter Assay

The cells were transfected by TurboFect Transfection Reagent (Thermo Scientific, Pittsburgh, PA, USA) according to the manufacturer’s instructions. The total amount of DNA for each experiment was matched to equal with their individual backbone vector. After transfection, the lysates of experimental cells were harvested for luciferase assay system following the manufacturer’s instructions (Promega, Madison, WI, USA). The 50 μL of cell lysate, 50 μL of luciferin, and 180 μL of luciferase assay reagent were injected into a luminometer tube. After votexing, the tube was placed in the luminometer and luciferase activity measured.

### Pre-miR-135a and Antisense of miR-135a (A-135a)-Inducible Stable Cells

The primers for PCR using pre-miR-135a forward (5′-*Age*I-AGGCCTCGCTGTTCTCTATGGC-3′) and pre-miR-135a reverse (5′-*Pme*I-TGTCCCCGCCGTGCG-3′) to generate pre-miR-135a construction in pAS4w.1.Pneo, a tetracycline-inducible system of lentiviral expression vector. The A-135a construction in pLAS1w.3xLacO, a lentiviral expression vector, used the DNA fragment as follows: 5′-TATGGCTTTTTATTCCTATGTGACTCGAGTCACATAGGAATAAAAAGCCATATTTTT-3′. Stable U373MG cells containing Pre-miR-135a and A-135a were generated by pLAS.AS3w.aOn.Pbsd lentiviral infectants and parental U373MG cells, respectively. Doxycycline (2 μg/mL) was used to induce miR-135a expression. IPTG (500 μM) was used to induce A-135a expression. The lentiviral expression vectors were obtained from the National RNAi Core Facility located at the Genomic Research Center of Institute of Molecular Biology, Academia Sinica (Taiwan).

### Western Blot Analysis

For Western analysis, cells were lysed with modified RIPA buffer [50 mM Tris-HCl (pH 7.4), 150 mM NaCl, 1 mM EDTA, 1 % Nonidet P-40, 0.25 % sodium deoxycholate, 1 mM PMSF, aprotinin 1 μg/mL, and leupeptin 1 μg/mL]. Lysates were resolved on a sodium dodecyl sulfate (SDS)-containing 10 % polyacrylamide gel, transferred to polyvinylidene difluoride (PVDF) nylon membrane and probed with anti-THBS1(1:1000), anti-CEBPD (1:2000), anti-α tubulin (1:10,000) at 4 °C overnight. Specific bands were detected by a horseradish peroxidase-conjugated antibody specific for either rabbit or mouse IgG (1:3000) and revealed by an enhanced chemiluminescence (ECL) Western blot system from Pierce (Rockford, IL, USA).

### Lentiviral Knockdown Assay

Virus was produced from Phoenix cells by co-transfection of the various shRNA expression vectors in combination with pMD2.G and psPAX2. After determining the viral infection efficiency, 10 M.O.I. of lentivirus containing shβ-galactosidase (shLacZ) or shCEBPD were used to infect U373MG cells for 48 h. In all lentiviral experiments, medium containing uninfected viruses was removed before conducting further assays. The shRNA sequences in lentiviral expression vectors shown as follows: shLacZ, 5′-CCGGTGTTCGCATTATCCGAACCATCTCGAGATGGTTCGGATAATGCGAACATTTTTG-3′ and shCEBPD, 5′-CCGGGCCGACCTCTTCAACAGCAATCTCGAGATTGCTGTTGAAGAGGTCGGCTTTTT-3′. The lentiviral knockdown expression vectors were obtained from the National RNAi Core Facility located at the Genomic Research Center of Institute of Molecular Biology, Academia Sinica (Taiwan).

### Chromatin Immunoprecipitation Assay

The chromatin immunoprecipitation (ChIP) assay was carried out essentially as described by Wang et al. [34]. Briefly, U373MG cells were treated with 1 % formaldehyde for 15 min. The cross-linked chromatin was then prepared and sonicated to an average size of 500 bp. The fragmented gDNA-protein complex was immunoprecipitated with antibodies specific for CEBPD or control rabbit immunoglobulin G at 4 °C overnight. After reversal of the cross-linking, the immunoprecipitated chromatin was amplified by primers related to the specific regions of the target genes genomic locus. The primers are as follows: FR-211 forward, 5′-TCCTCTACTGGCCCTTTAGTCCCTG-3′ and FR-211 reverse, 5′-AGCTGGTTCTCAGCTGAAACAAAGA-3′; FR-250 forward, 5′-AGAGACAGAGACAGATAGGAAGCAA-3′ and FR-250 reverse, 5′-ACAGAAAAGTTGCCAGAATAGTGAT-3′. The amplified DNA products were resolved by agarose gel electrophoresis.

### In Situ Hybridization

Non-radioactive in situ hybridization was performed on 10 μm sections from mouse brain tissue blocks using the digoxigenin-labeled locked nucleic acid-modified detection probe (20 μM; 5′-TCACATAGGAATAAAAAGCCATA-3′; Exiqon, Tustin, CA) complementary to mature miR-135a and IsHyb in situ hybridization kit (BioChain, Hayward, CA) following the protocol from Exiqon. Briefly, 10 μm sections were deparaffinized, hydrated, and treated with proteinase K (10 μg/mL; QIAGEN) at 37 °C for 15 min and fixed with 4 % paraformaldehyde for 20 min. The sections were incubated in pre-hybridization solution at 50 °C for 3 h and then in hybridization solution with digoxigenin-labeled, locked nucleic acid-modified miR-135a probe at 50 °C for 16 h. The sections were incubated in the phosphate-buffered saline diluted anti-digoxigenin-AP antibody (1:100) at room temperature for 1 h, washed with 2× standard saline citrate (SSC), 1.5× SSC, 0.2× SSC, incubated in blocking solution for 1 h at room temperature, washed with phosphate-buffered saline three times, then washed with 1× alkaline phosphatase buffer twice. The NBT/BCIP solution was used for visualization of hybridization signal.

### Treatment of *App*Tg Mice with Antagomirs

Mice were anesthetized by intraperitoneal injection of chloral hydrat (4 mg/kg) and then positioned in a stereotaxic apparatus. Using a 30-guage Hamilton syringe, 1 μL of phosphate-buffered saline containing 0.5 nmol of the antagomir Cy3-labeled AM135a or a scrambled antagomir (RiboBio ,Guangzhou, China) was injected over 5 min into the third ventricle at the following coordinates: antero-posterior, −1.06 mm; medio-lateral, 0.00 mm; and dorso-ventral, −2.4 mm from the bregma. The needle was left in place for another 10 min and then gently removed.

### Morris Water Maze Test

The training apparatus was a circular white pool (120 cm in diameter) containing water at 24 °C. A platform (10 cm in diameter) was submerged 1 cm under the water surface. Before the test, mice were first trained to swim and climb on to the platform. For the hidden platform trial, four sessions were performed (four trials per session per day were carried out). Mice were allowed to search for the platform for 120 s. If the mice did not find the platform within 120 s, they were gently guided to it. All mice were allowed to remain on the platform for 20 s. The time spent to reach the hidden platform was recorded.

## Results

### Role of Astrocytic CEBPD in Neuron Apoptosis and Spatial Learning in *App*Tg Mice

**Fig. 1:**
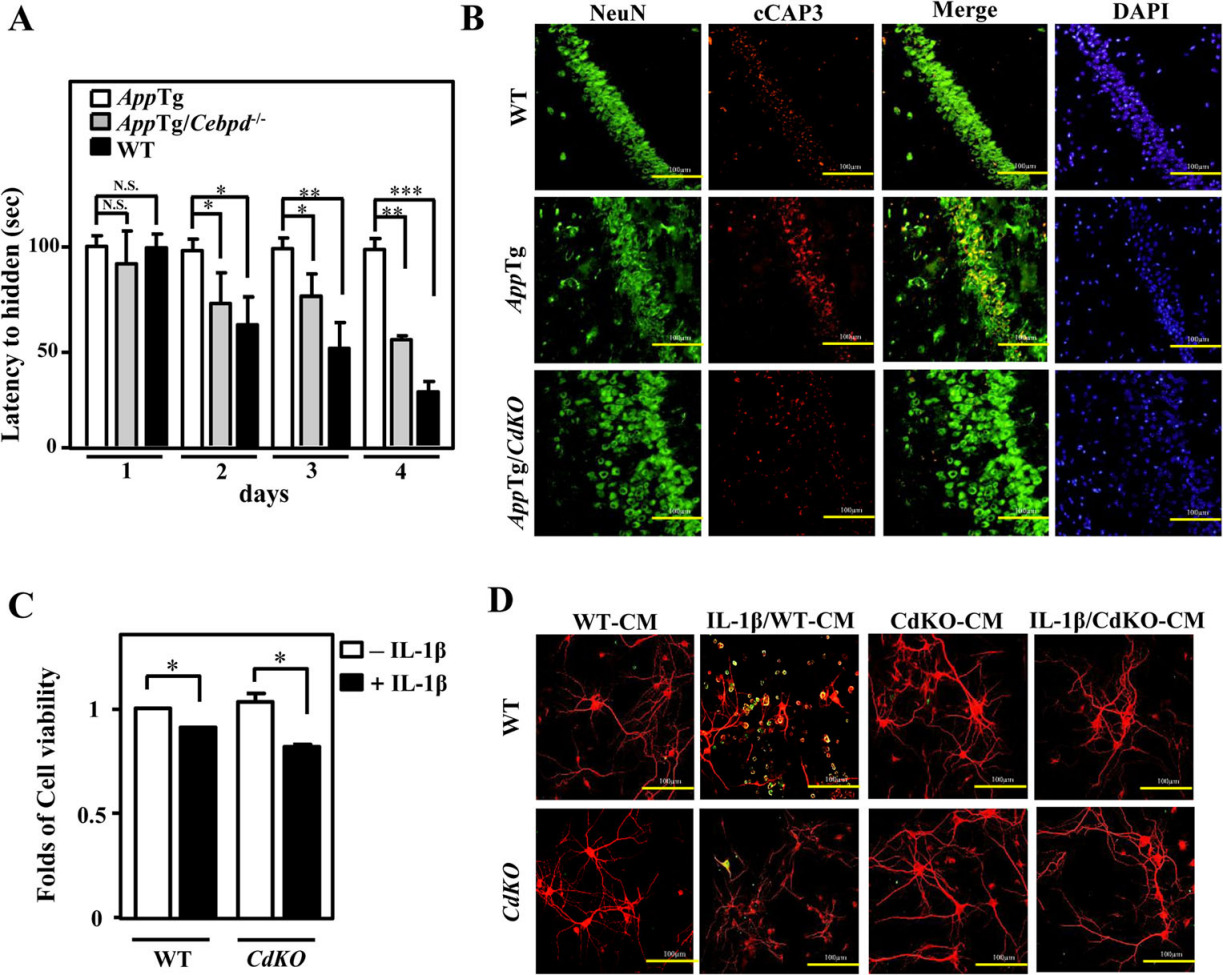
Neuronal apoptosis and spatial learning ability in *App*Tg/*Cebpd* ^−/−^ mice. **a** Spatial learning ability of wild-type (WT; C57BL/6), *App*Tg, and *App*Tg/*Cebpd* ^−/−^ mice. Time required by WT (*n* = 5), *App*Tg (*n* = 5), and *App*Tg/*Cebpd* ^−/−^ (*n* = 3) mice to reach the hidden platform (latency to hidden) was analyzed in the Morris water maze. **b** The apoptosis of neurons was attenuated in *App*Tg/*Cebpd* ^−/−^ mice. The brain tissues were subjected to immunofluorescence staining with the antibody recognized NeuN or cleaved caspase 3 (cCap3; active caspase 3). *Scale bar* = 100 μm. **c** Primary *Cebpd* ^−/−^ neurons have no effect to reverse IL-1β-induced cell death. The MTT assay was conducted as indicated. (**d**) Attenuated *Cebpd* expression in primary astrocytes does not contribute to neuronal death. The conditioned medium came from either IL-1β-pretreated primary WT or *Cebpd* ^−/−^ astrocytes. Primary cortical neurons in WT and *Cebpd* ^−/−^ mice were seeded and grown on coverslips with the mixture medium (9:1) of neuronal maintenance medium and above conditioned medium for 72 h. The experimental neurons were performed with immunofluorescence staining by anti-MAP2 antibodies for morphological examination and anti-cleaved caspase 3 (cCap3) antibodies for cell death detection. *Scale bar* = 100 μm. Data represent two independent experiments with three mice/group in B or three independent experiments in **c** and **d**. Data are expressed as mean ± SEM by a Student’s *t* test. (*P < 0.05; **P < 0.01; ***P < 0.001; *NS* not significant)

To find out whether the expression of Cebpd has direct involvement in the spatial learning ability of mice, Morris water maze was conducted using wild-type (*Cebpd* ^+/+^), *AppTg*, and *AppTg*/*Cebpd* ^−/−^ mice. As expected, *AppTg* mice have impaired spatial ability and failed to show improvement in latency to hidden even on day 4 (Fig. 1a). Interestingly, *AppTg*/*Cebpd* ^−/−^ mice showed a time-dependent improvement in their ability to navigate through the water maze. However, *AppTg*/*Cebpd* ^−/−^ mice’s improvement in latency to hidden was not as pronounced as wild-type mice. To find out whether neuron viability has a role in *AppTg*/*Cebpd* ^−/−^ mice’s improvement in latency to hidden, we compared the staining of caspase 3 in *AppTg* and *AppTg*/*Cebpd* ^−/−^ mice. As compared to *AppTg* mice, the staining of caspase 3 was greatly attenuated in *AppTg*/*Cebpd* ^−/−^ mice (Fig. 1b). To quantitatively determine if *Cebpd* has a role in inflammation-dependent cell viability, MTT assay was conducted in primary *Cebpd* ^−/−^ neurons treated with IL-1β. To our surprise, the primary *Cebpd* ^−/−^ neurons did not enhance their cell viability as compared to primary *Cebpd* ^+/+^ neurons, upon IL-1β treatment (Fig. 1c). Thus, we tested whether astrocytic CEBPD was responsible for the viability of neuron instead. To address this issue, conditioned media of IL-1β-pretreated primary wild-type or *Cebpd* ^−/−^ astrocytes were incubated with primary neuron cells. The survival and neurite outgrowth of neurons showed little difference between *Cebpd* ^−/−^ and wild-type neurons, if IL-1β pretreated CM from wild-type astrocytes were used. However, upon incubation of the CM from IL-1β-pretreated primary *Cebpd* ^−/−^ astrocytes, the survival and neurite outgrowth of *Cebpd* ^−/−^ neurons were attenuated (Figs. 1d and S1).

## CEBPD Represses THBS1 Transcription in Astrocytes

**Fig. 2:**
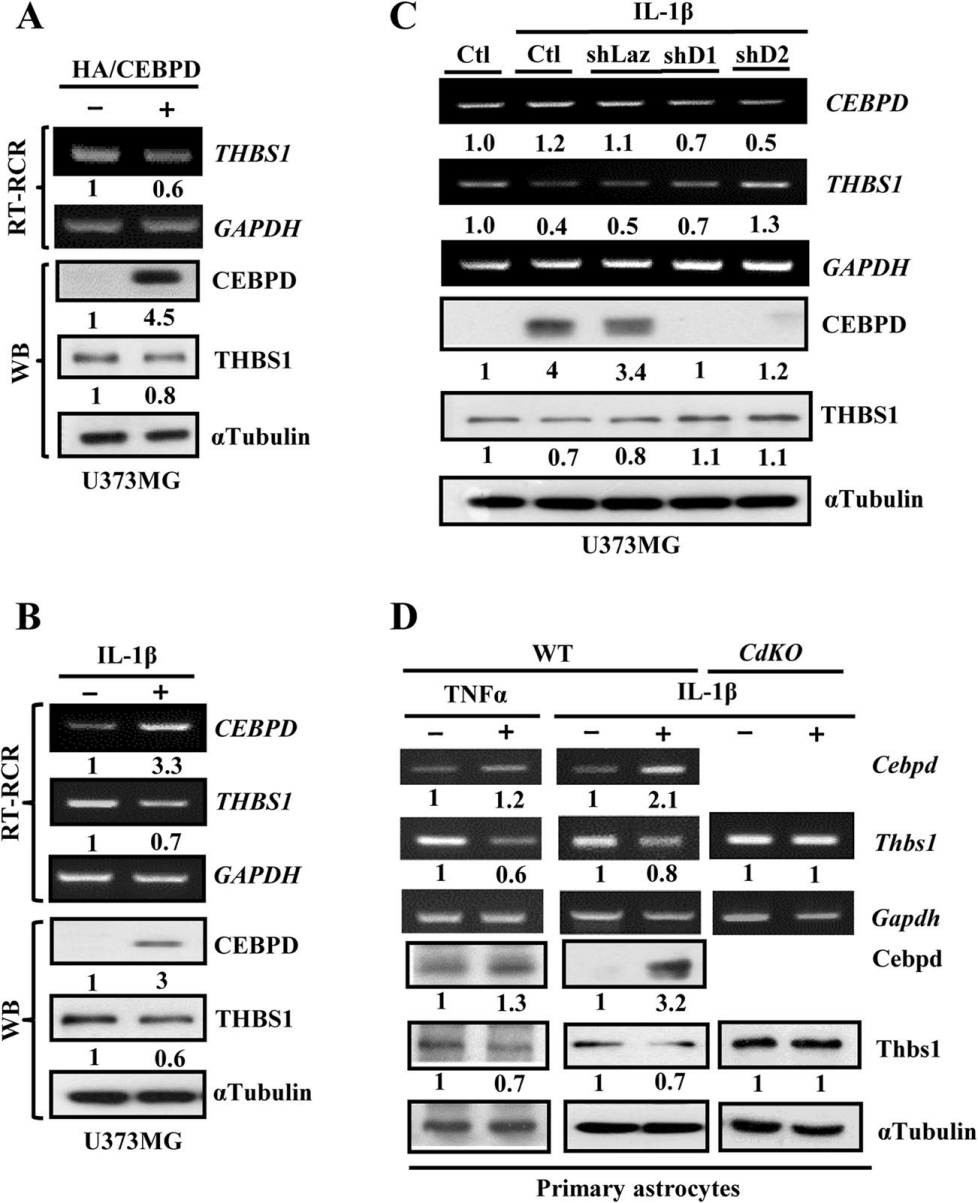
*THBS1* transcription is repressed by CEBPD in astrocytes. **a** CEBPD contribute to THBS1 mRNA and protein degradation. U373MG cells stably transfected with the zinc-inducible CEBPD expression vector and then incubated in the presence or absence of 100 μM ZnSO_4_ for 6 h. RT-PCR and Western blot analysis were performed to examine the expressions of THBS1 mRNA and protein. **b** THBS1 expression is suppressed after IL-1β treatment. U373MG cells were treated with 5 ng/mL IL-1β for 3 h and then RT-PCR assay and Western blot analysis were performed to examine the expressions of THBS1 mRNA and protein, respectively. **c** CEBPD participates in IL-1β repressed THBS1 transcription. U373MG cells were infected with lentivirus of sh-galactosidase (shLacZ) or shCEBPD (shD1, shD2) and then treated with IL-1β for 3 h. Total RNA or cell lysates were harvested for RT-PCR or Western blot analysis. **d** Loss of CEBPD in primary astrocytes reverses IL-1β-attenuated Thbs1 expression. Primary *Cebpd* ^*+/+*^ (WT) and *Cebpd* ^−*/*−^ (CdKO) astrocytes were treated with 20 ng/mL TNFα, or 5 ng/mL IL-1β for 3 h, then the RT-PCR and Western blot were performed to examine the expression of Thbs1 mRNA and protein, respectively

Astrocyte-secreting neurotrophic factors are essential for neuronal survival and synaptic plasticity. Our global profiling assay showed that *THBS1*, a neurotrophic factor, was repressed in U373MG cells expressing CEBPD (Table 1). The possibility that CEBPD can directly repress the expression of THBS1 in U373MG cells was tested by transfection with the zinc-inducible CEBPD expression vector. RT-PCR and Western blot studies found that overexpression of CEBPD lead to decrease in expression of THBS1 (Fig. 2a). Thus, we looked at whether IL-1β treatment has an effect on THBS1 expression. Consistently, it was found that IL-1β induced expression of CEBPD, leading to decrease in THBS1 expression (Fig. 2b). To find out whether CEBPD depletion can lead to an opposite effect as compare to CEBPD overexpression, IL-1β-treated U373MG cells were infected with two different shRNA against CEBPD. It was found that knock down of CEBPD can lead to increase in *THBS1* expression instead (Fig. 2c). Finally, the THBS1 repressive effect of CEBPD was examined in mouse primary astrocytes (Fig. 2d). Treatment of mouse primary astrocytes with either TNFα or IL-1β can both lead to increase expression of CEBPD and a decrease in expression of THBS1. Interestingly in *Cebpd* ^−/−^ primary astrocytes, it was observed that there is a constantly high expression of Thbs1.

**Table 1:** Genes in response to CEBPD induction and CEBPD responsive miRNAs in U373MG cells

Upregulated miRNAs	Folds of change	Downregulated mRNAs	Folds of change	Downregulated miRNA	Folds of change	Upregulated mRNA	Folds of change
hsa-mir-135a	1.6	THBS1	0.57	hsa-miR-30c	0.59	ME1	1.64
		SERPINC1	0.1	hsa-miR-24	0.62	ADAMTS 5	1.54
hsa-mir-1254	1.6	ETV7	0.55			BCL2L11	1.54
hsa-mir-1321	1.7	BAX	0.65			PDGFRA	1.51
		BCL2L1	0.62	hsa-miR-17	0.64	ETV1	1.53
		SYK	0.6	hsa-miR-320	0.64	ETV1	1.53
		DTX1	0.65			KITLG	1.85
hsa-mir-198	1.74	DTX	0.65	hsa-miR-320b	0.64	ETV1	1.53
						KITLG	1.85
				hsa-miR-320c	0.64	ETV1	1.53
						KITLG	1.85
				hsa-miR-16	0.65	ADAMTS5	
						ITGA2	1.68
				hsa-miR-29b	0.65	BMF	1.51
						PTX3	1.86
				hsa-miR-15b	0.66	ADAMTS5	1.54
						ITGA2	1.68
				hsa-miR-181b	0.68	ADAMTS5	1.54
						KITLG	1.85
						PDGFRA	1.51
						PRLR	1.98
				hsa-miR-19b	0.68	ABCA1	1.89
						ID2	1.51
				hsa-miR-23a	0.68	CA2	1.92
						SLC1A1	1.56
						TNFAIP6	1.65
				hsa-miR-29a	0.68	BMF	1.51
						PTX3	1.86
				hsa-miR-29a	0.69	RGS2	1.91
				hsa-miR-29a	0.69	RHOU	1.61

## CEBPD Activates miR-135a Transcription

**Fig. 3:**
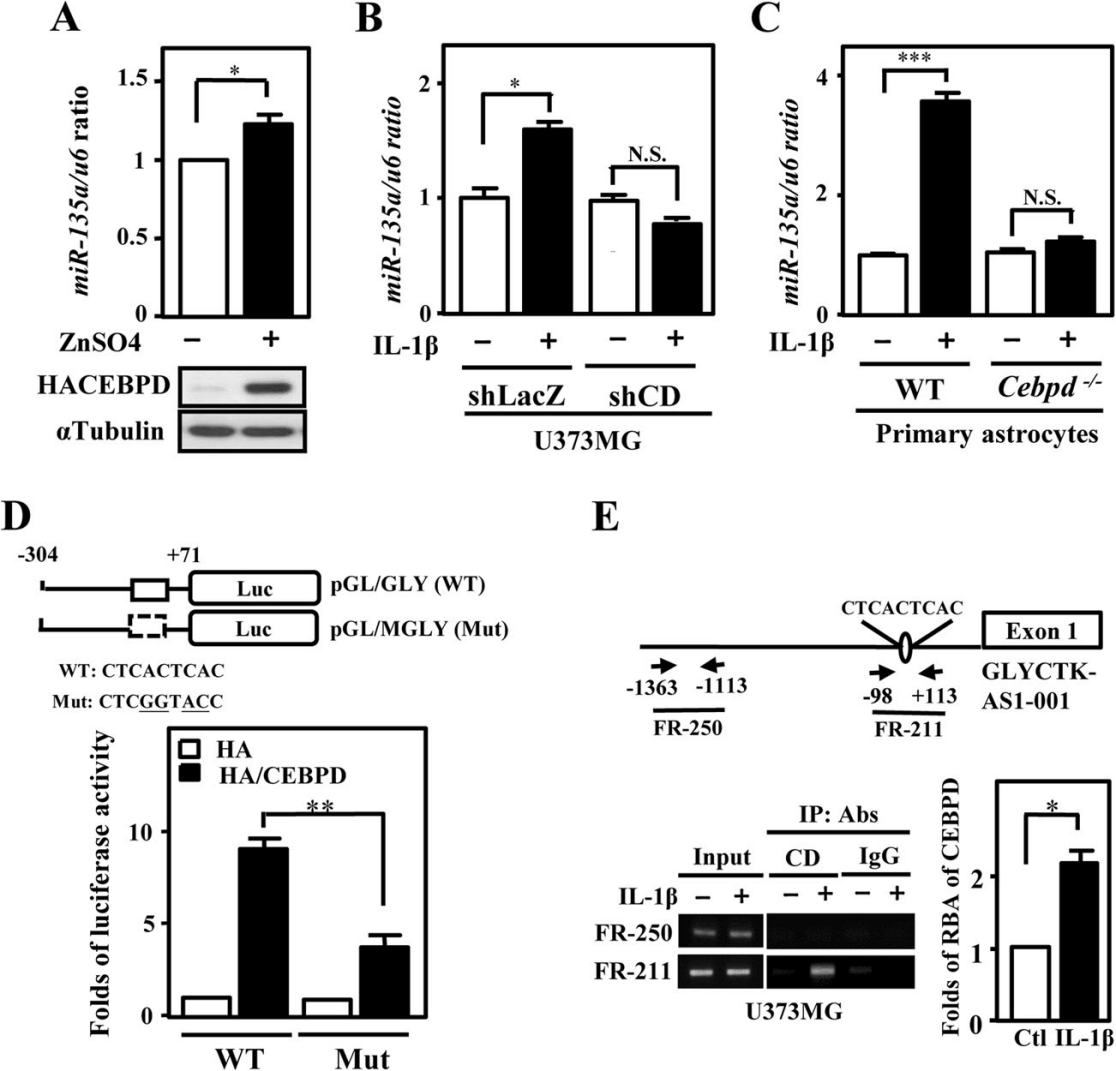
miR-135a is a CEBPD responsive miRNA. **a** CEBPD induces miR-135a expression. qRT-PCR and Western blot confirmed that miR-135a levels and the expression of HA-tagged CEBPD protein from stable U373MG cells with pMT-CEBPD expression vector, respectively. **b** CEBPD participate in IL-1β-induced miR-135a expression. qRT-PCR was performed with total RNA harvested from IL-1β-treated U373MG cells with or without attenuation of CEBPD. **c** miR-135a expression is unaltered in primary *Cebpd* ^−*/*−^ astrocytes. qRT-PCR was performed using total RNA harvested from primary *Cebpd* ^*+/+*^ and *Cebpd* ^−*/*−^ astrocytes with or without IL-1β treatment. **d** CEBPD increases miR-135a promoter activities. Schematic representation of reporter constructs with the GLYCTK-AS1-001 promoter. The approximate location of putative CEBPD-binding motif was indicated by *open box*. A luciferase activity was conducted by co-transfected reporter and expression vectors as indicated in U373MG cells. **e** CEBPD can directly bind to the GLYCTK-AS1-001 promoter in vivo. A ChIP assay was performed with U373MG cells treated with IL-1β. Chromatin of U373MG cells was separately immunoprecipitated with specific antibody against CEBPD (CD) and control IgG [14]. The schematic on the top indicated the location of the primers used for detection of the GLYCTK-AS1-001 promoter by PCR. The precipitated DNAs were amplified by PCR with primer (FR-211) on the GLYCTK-AS1-001 promoter (−98/ +113) which contain putative CEBPD-binding motif and negative control primer (FR-250). The data represented the mean ± standard error of three independent experiments, each performed in triplicate. (*P < 0.05, **P < 0.01, ***P < 0.001, Student’s *t* test)

To explore which miRNAs are regulated by CEBPD in astrocytes, we conducted a microarray profiling to identify the CEBPD-regulated miRNAs (Table 2). Interestingly, miR-135a, a putative *THBS1* mRNA binding suppressor, was observed to be upregulated by CEBPD in the global profiling of miRNA. To find out if CEBPD expression has direct correlation with miR-135a expression, we investigated the expression of miR-135a in U373MG cells either by overexpression of CEBPD or knock down of CEBPD. We obtained a consistent result in which the overexpression of CEBPD results in increasing expression of miR-135a (Fig. 3a), while knock down of CEBPD results in reduce expression of miR-135a upon IL-1β stimulation (Fig. 3b). Subsequently, we explored whether CEBPD still regulate miR-135a in primary astrocytes. It was observed that the expression of miR-135a still increased upon IL-1β treatment in primary *Cebpd* ^+/+^ but not in *Cebpd* ^−/−^ astrocytes (Fig. 3c). Next, we wished to find out how CEBPD can increase the expression of miR-135a. Since miR-135a is located at intron 1 of *GLYCTK*-*AS1*-*001* gene, we first made sure that increase expression of CEBPD can indeed induce the expression of *GLYCTK*-*AS1*-*001* transcripts (Fig. S2). We conducted a luciferase activity assay and found that the native *GLYCTK*-*AS1*-*001* reporter was activated by CEBPD (Fig. 3d). However, the luciferase activity of the mutated *GLYCTK*-*AS1*-*001* reporter was attenuated in contrast to native one. Thus, we looked at whether CEBPD can directly bind to the promoter of *GLYCTK*-*AS1*-*001* by a chromatin-IP (ChIP) assay (Fig. 3e). The result showed that indeed CEBPD can bind to the region corresponding to our primers.

**Table 2:** CEBPD responsive miRNAs in U373MG cells

Upregulated	Fold of change	Downregulated	Fold of change
hsa-miR-4314	2.2	hsa-miR-22	0.7
hsa-miR-788	1.9	hsa-miR-1979	0.7
hsa-miR-557	1.8	hsa-miR-200b	0.7
hsa-miR-198	1.7	hsa-miR-26b	0.7
hsa-miR-2116	1.7	hsa-miR-181b	0.7
hsa-miR-1321	1.7	hsa-miR-29a	0.7
hsa-miR1193	1.7	hsa-miR-138	0.7
hsa-miR-4299	1.7	hsa-miR-23a	0.7
hsa-miR-3131	1.6	hsa-miR-19b	0.7
hsa-miR-3190	1.6	hsa-miR-423	0.7
hsa-miR-4300	1.6	hsa-miR-515	0.7
hsa-miR-135a	1.6	hsa-miR-128	0.7
hsa-miR1254	1.6	hsa-let-7c	0.7
hsa-miR-514b	1.6	hsa-miR-1975	0.7
hsa-miR-1909	1.6	hsa-miR-708	0.7
hsa-miR-921	1.6	hsa-miR-15b	0.7
hsa-miR-3144	1.6	hsa-miR-103	0.7
hsa-miR-3125	1.5	hsa-miR-720	0.7
hsa-miR-1914	1.5	hsa-miR-16	0.7
hsa-miR-4257	1.5	hsa-miR-29b	0.7
hsa-miR-4270	1.5	hsa-miR-1201	0.7
		hsa-miR-320c	0.6
		hsa-miR-17	0.6
		hsa-miR-320a	0.6
		hsa-miR-320b	0.6
		hsa-miR-21	0.6
		hsa-miR-9	0.6
		hsa-miR-196a	0.6
		hsa-miR-24	0.6
		hsa-miR-107	0.6
		hsa-miR-1248	0.6
		hsa-miR-30c	0.6
		hsa-miR-455	0.6

## miR-135a Can Target to *THBS1* (*Human)/Thbs1 (Mouse)* 3′UTR and Contributes to the Repression of THBS1/ Thbs1

**Fig. 4:**
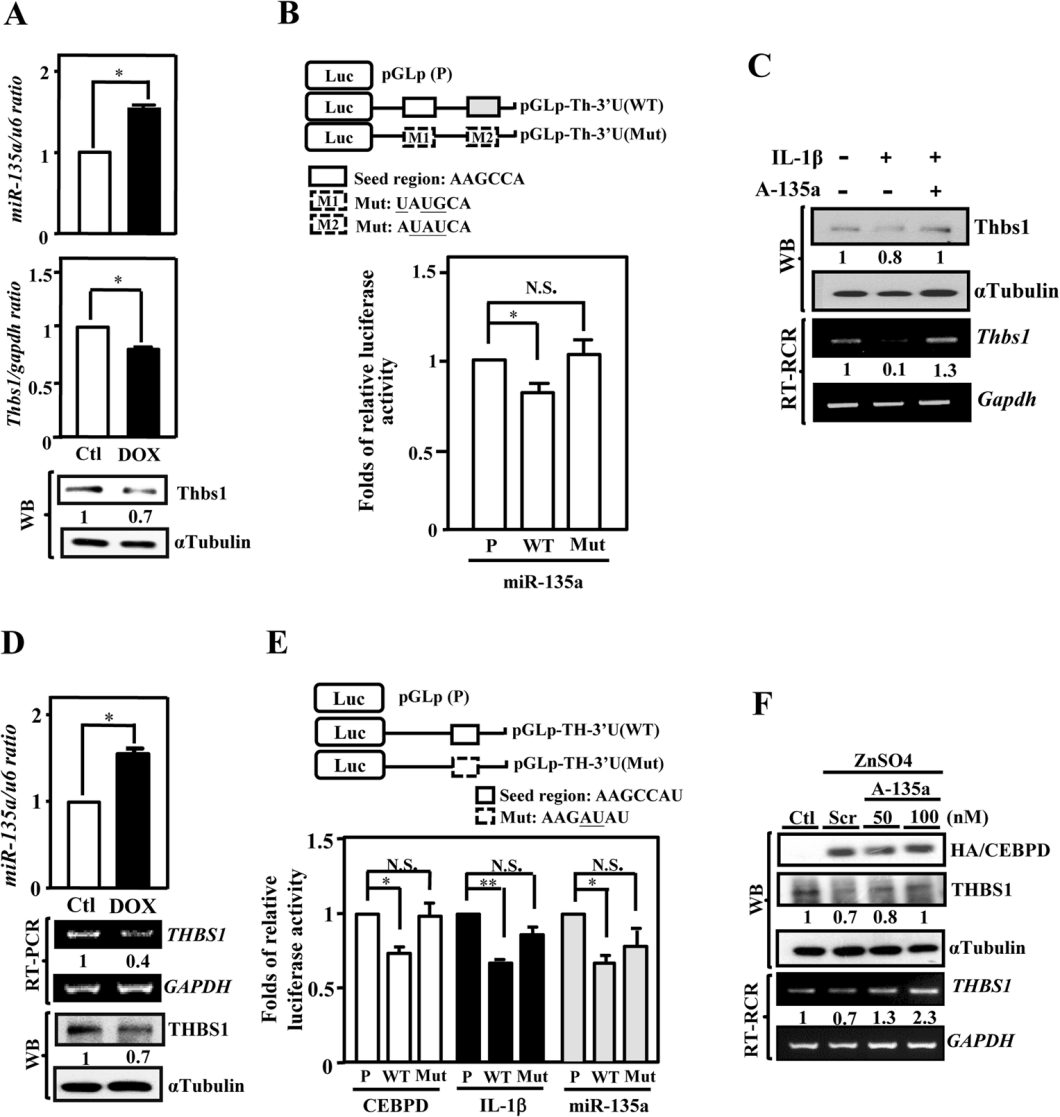
miR-135a suppresses Thbs1/THBS1 transcription through its 3′UTR region. **a** miR-135a attenuates the expression of Thbs1. Induced miR135a in primary astrocytes with DOX-inducible miR-135a expression system; qRT-PCR and western blot analysis confirmed that miR-135a, Thbs1 mRNAs, and proteins levels, respectively. **b** Two positions of the Thbs1 3′-untranslated region are predicted to be targets of miR-135a. The seed regions were indicated by the open box (upper panel). Luciferase activity of reporter constructs was measured after co-transfected with pre-miR-135a. **c** Antisense of miR135a (A-135a) antagonize the effects of Cebpd. Induced A-135a in primary astrocytes with IPTG-inducible A-135a expression system and treated with or without IL-1β treatment. **d** Induced miR-135a expression repress the THBS1 expression. In stable U373MG cells with DOX-inducible miR-135a expression system, the expression of miR-135a and the level of THBS1 mRNAs and proteins were examined by qRT-PCR, RT-PCR, and Western blot, respectively. **e** IL-1β and CEBPD suppresses THBS1 transcription via miR-135a binding motif. The seed region was indicated by the *open box* (upper panel). Luciferase activity of reporter constructs was measured after co-transfected with pre-miR-135a, CEBPD expression vectors, or treated IL-1β. **f** Antagomir of miR135a (AM135a) dose-dependently antagonized the effects of CEBPD. AM135a or scramble antagomir were transfected into the U373MG stable cells with zinc-inducible CEBPD expression system and then incubated in the presence or absence of 100 μM ZnSO4 for 6 h. RT-PCR and Western blot analysis were performed to examine the expressions of THBS1 mRNA and protein. The data represent the mean ± standard error of three independent experiments, each performed in triplicate. (*P < 0.05, **P < 0.01, Student’s *t* test)

To verify that miR-135a can indeed repress the expression of THBS1, we infected a DOX-inducible miR-135a expression system into primary astrocytes (Fig. 4a) and U373MG cells (Fig. 4d). In both studies, we found that increase expression of miR-135a attenuates the level of THBS1. Our recent study demonstrated that prostaglandin 2 (PGE2) could repress *THBS1* 3′UTR reporter activity through a miR-135a binding motif. Two miR-135a binding motifs were identified in *Thbs1* 3′UTR. To confirm that *Thbs1* 3′UTR is indeed targeted by miR-135a, we constructed luciferase reporter plasmids that contained native or mutated seed sequences of *Thbs1* 3′UTR. The reporter assay were conducted by co-transfecting various mouse *Thbs1* 3′UTR reporters with miR-135a expression vector. We found that miR-135a repressed the native *Thbs1* 3′UTR reporter activities, but these effects were lost in construct with individual mutations of miR-135a binding motifs (Fig. 4b). Similar results were also obtained from co-transfecting various *THBS1* 3′UTR reporters with CEBPD, pre-miR-135a expression vectors, or IL-1β stimulation (Fig. 4d). Next, we investigated whether inhibition of miR-135a can lead to the opposite effect of increasing THBS1 expression. We found that expression of antisense of miR-135a (A-135a) in primary astrocytes (Fig. 4c) or miR-135a antagomir (AM135a) in U373MG cells (Fig. 4f) can lead to increase THBS1 expression. Interestingly, a dose-dependent increased in expression of CEBPD was also found upon AM135a expression in U373MG cells.

## Attenuated CEBPD and miR-135a in Astrocytes Restores the Neuronal Neurite Outgrowth

**Fig. 5:**
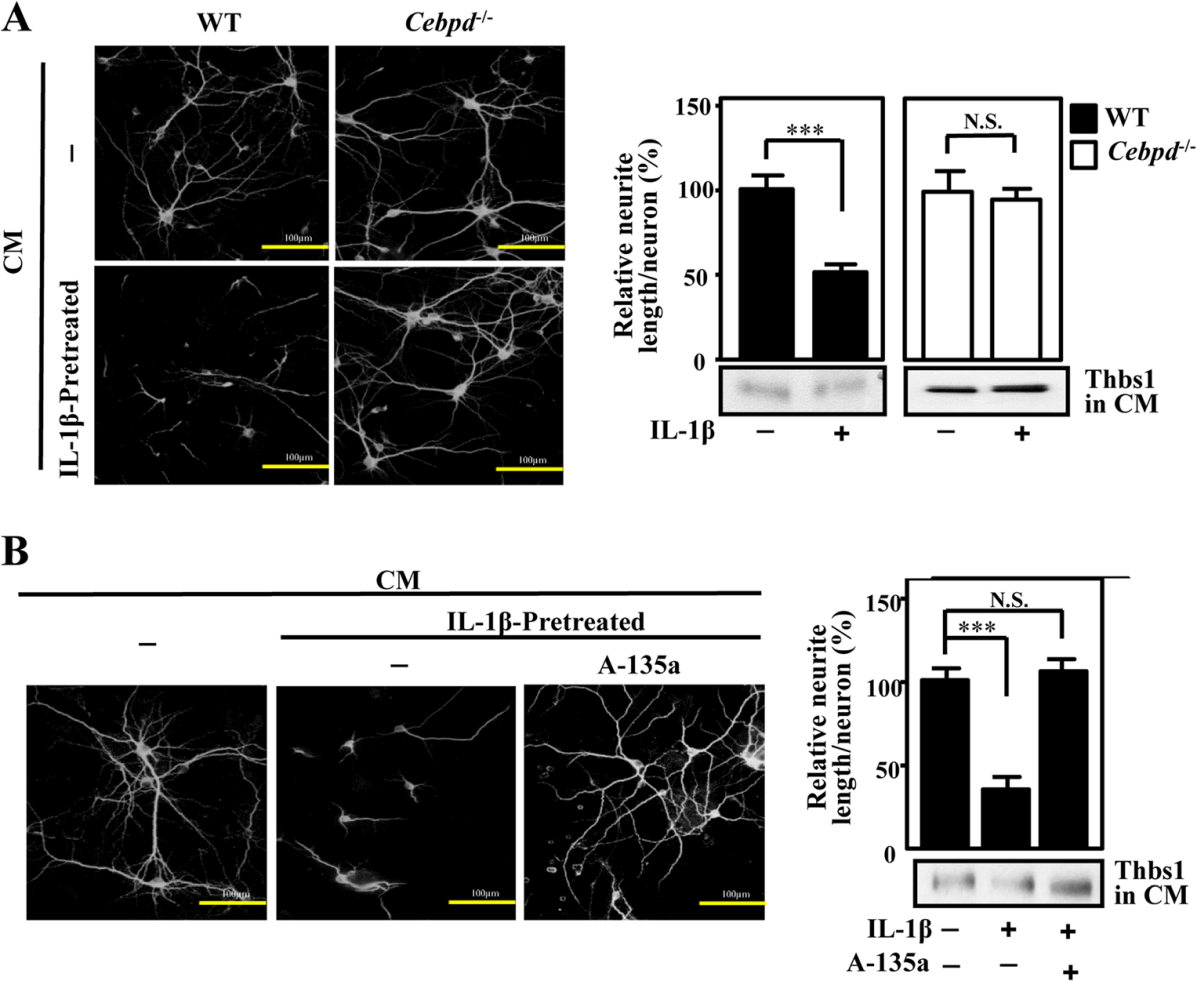
Astrocytic CEBPD and miR-135a regulate the extension of primary cortical neurons. **a** CEBPD in astrocytes reduces neuronal neurite length. Conditioned medium from primary *Cebpd* ^*+/+*^ or *Cebpd* ^−*/*−^ astrocytes with or without IL-1β treatment were subjected to exam Thbs1 by Western blot and added to primary neurons for 48 h. **b** Attenuated miR-135a in astrocytes restore the neuronal neurite outgrowth. Conditioned medium from primary astrocytes infected with IPTG-inducible A-135a expression system with or without IL-1β treatment were subjected to exam Thbs1 by Western blot and added to primary neurons for 48 h. Neurons were stained with mouse anti-MAP2 Abs for morphological examination. *Scale bar* = 100 μm. ImageJ software was used to quantification micrometers of neurite length /neuron. Quantification of relative of neurite length/neuron. (*P < 0.05, **P < 0.01, Student’s *t* test)

It has been found that the growth of neurite decrease during inflammation. We wished to determine whether knock out of CEBPD in astrocyte could still lead to growth of primary neurons upon IL-1β treatment. Conditioned medium from primary *Cebpd* ^*+/+*^ or *Cebpd* ^−*/*−^ astrocytes with or without IL-1β treatment was added to primary neurons for 48 h. It was found that neurons showed a reduction in length upon incubation with CM containing *Cebpd* ^*+/+*^ astrocytes with IL-1β treatment, but there were no changes in length of primary neurons upon incubation with CM containing *Cebpd* ^−*/*−^ astrocytes with IL-1β treatment (Fig. 5a). Consistently, we also found that there is reduction in the length of neurons when incubated with CM containing either U373MG cells overexpressing either CEBPD or miR-135a (Fig. S3a, S3b). There is also no change in the length of primary neurons upon incubation with CM containing either astrocytes (Fig. 5b) or U373MG cells (Fig. S3c) expressing antisense of miR-135a (A-135a).

## Intracerebral Injection of miR-135a Neutralizing Antagomir Decreases Neuronal Apoptosis and Enhances Spatial Learning Ability in AppTg Mice

**Fig. 6:**
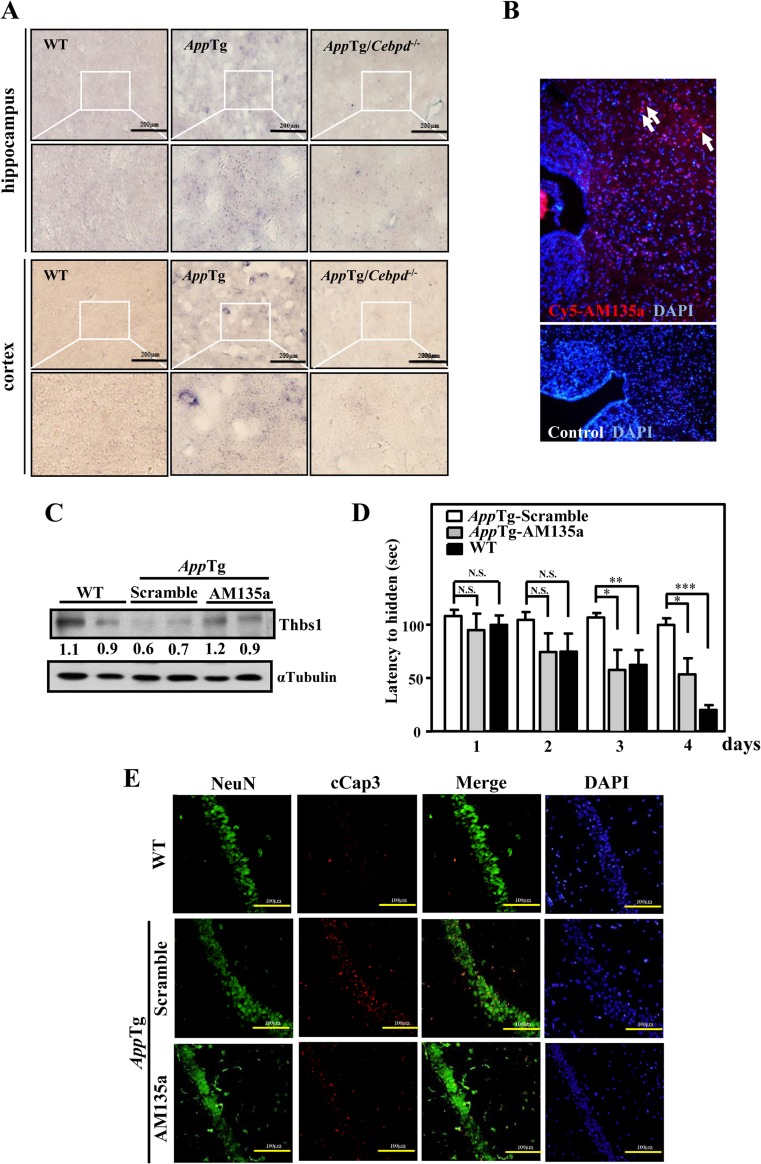
The *in vivo* effects of AM 135a. **a** miR-135a expression increase in the brain tissues of *AppTg* mice. miR-135a signal was detected by in situ hybridization in the hippocampus and cortex of WT, *AppTg*, and *AppTg/Cebpd* ^−*/*−^ mice. *Scale bar* = 200 μm. **b** Cy3-AM135a efficiently injected into *AppTg* mice. Twelve-month-old *AppTg* mice were injected in the third ventricle with Cy3-labeled AM135a. At 24 h, fluorescence for Cy3-AM135a was distributed throughout the hippocampus and surrounding tissues. Cy3 fluorescence was not detected in control (noninjected) brains. Insets show magnified views of the regions indicated with *arrows*. Nuclei were stained with DAPI. **c** AM135a significantly increased the whole brain level of Thbs1. Western blot analysis conducted to detect Thbs1 of *App*Tg mice injected with AM135a and the scramble antagomir at 1 week after the injection. **d** AM135a improved the spatial learning ability of *App*Tg mice. Intraventricular injected *App*Tg mice of AM135a (AppTg-AM135a) or scramble antagomir (AppTg-Scramble). Morris water maze test for spatial learning were performed 1 week after injection. Time required by WT (*n* = 5), AppTg-AM135a (*n* = 5), and *AppTg*-Scramble (*n* = 5) mice to reach the hidden platform (latency to hidden) were analyzed. **e** Cleavage caspase 3 (cCap3) expression was reduced in *App*Tg mice treated with AM135a. The brain tissues were subjected to immunofluorescence with anti-NeuN and anti-cleaved caspase 3 (cCap3). *Scale bar* = 100 μm. Data are expressed as mean ± SEM by a Student’s *t* test. (*P < 0.05; **P < 0.01; ***P < 0.001; *NS* not significant)

We wished to determine whether AM135a could be used as a therapeutic agent on *App*Tg mice. First, we investigated the expression of miR-135a in mouse brain by in situ hybridization. Enhanced expression of miR-135a was found in the brain of a 12-month-old *AppTg* mice but not in *AppTg/Cebpd* ^−*/*−^ mice (Figs. 6a and S4). This result prompted us to inject a Cy3-labeled AM135a (Cy3-AM135a) into the third ventricle of the 12-month-old *App*Tg mice. We found that Cy3-AM135a was distributed throughout the hippocampus and surrounding tissue after 24 h (Fig. 6b). Consistent with our previous studies, the whole brain level of Thbs1 showed increased in expression at 1 week after the intraventricular injection of 0.5 nmol AM135a (Fig. 6c). Next, we measured the effect of AM135a on preventing neuronal apotoposis. It was found that in brain section from *AppTg* mice injected with AM135a, activated caspase 3 expression was attenuated and co-localized with NeuN-positive markers (Fig. 6e). The caspase activity of brain lysates from *AppTg* mice injected with AM135a were also attenuated (Fig. S5). Finally, we carried out Morris Water Maze test on *AppTg* mice treated with AM135a. The escape latency of *AppTg* mice treated with AM135a was significantly decreased after day 3 when it was compared with mice treated with scrambled antagomir (Fig. 6d).

## Discussion

AD is a common neurodegenerative disease, which is associated with neuronal loss and severe cognitive deficit. Most studies about AD have focused on neuronal dysfunction, but few have concentrated on astrocytes’ role in pathogenesis of AD. In AD patients and *AppTg* mice, CEBPD and Cebpd, respectively, are found to be highly expressed in astrocytes surrounding Aβ plagues [26, 20]. We found that *AppTg*/*Cebpd* ^−/−^ mice showed a spatial learning improvement compared with the *AppTg* mice. The *Cebpd*-deficient neuron was not found to play a role in inflammation-induced apoptosis. Since astrocytes are critical glial cells to support CNS by providing neurotrophic factors, we therefore tested whether certain neurotrophic factors were lost in activated astrocytes in response to proinflammatory factors. The neurotrophic factor THBS1 secreted from astrocytes was found to be attenuated in IL-1β-treated astrocytes. We also found that the activation of miR-135a transcription was responsive to transcription factor CEBPD and represses THBS1 abundance in astrocytes. In addition to our recent discovery that miR-135a plays an angiogenic role [27], we further demonstrated that astrocytic miR-135a participates in inhibition of neuronal viability and spatial learning ability in *App*Tg mice. We found that *App*Tg mice treated with AM135a had less dead neurons and showed improvement in spatial learning ability.

*Cebpd* ^−/−^ mice are viable and have normal spatial learning ability as compared to wild-type mice [35]. However, the loss of Cebpd was found to delay the progression and pathogenesis of some inflammatory diseases [23, 25]. It implies that CEBPD may not be essential in normal development, but can play important roles in the pathogenesis of inflammatory diseases. However, the details of CEBPD biology in inflammation and inflammation-associated diseases remain largely uninvestigated. Inflammation has been associated with many neuronal diseases including AD, Parkinson’s disease (PD), spinal cord injury, amyotrophic lateral sclerosis, and multiple sclerosis [1]. Common physiological consequents including the memory loss, dementia, and cognitive impairment have been observed in many neuroinflammatory diseases such as AD, Parkinson’s and Huntington’s disease (HD) [36]. However, as mentioned above, the crosstalks, especially in response to the activation of astrocytic CEBPD, between astrocytes and neurons in the inflamed brains have not been characterized.

Previously, we provided evidences to demonstrate that the activation of astrocytic CEBPD can contribute to inhibition of phagocytosis, promotion of microglia migration, and chemoattraction [26, 24]. The reduced extracellular level of astrocytic THBS1 could be caused by amyloid-beta (Aβ) and in Down syndrome, a disorder with a strong predilection for the development of AD [37, 38]. Our study showed a coincident result that the THBS1 abundance is attenuated in AD patients [39]. Accordingly, decreased neuronal neurite outgrowth, survival, and synaptophysin levels were also reported to be associated with both knock down or knock out of THBS1 [40, 37]. We found that the activation of astrocytic CEBPD contributed to repression of neurotrophic factor THBS1 abundance, which could result in the shortened neurite length and death of neurons. Moreover, we revealed that the CEBPD-upregulated miR-135a can target to *THBS1*-3′UTR and resulted in its mRNA and protein degradations.

Dystrophic neurites are a hallmark in the brain of AD and associated with mental decline and dementia [41]. Previous studies showed that Aβ contribute to neurite degeneration in cultured neurons and ultimately result in neuronal death [42, 43]. In addition to proinflammatory cytokines IL-1β and TNFα, the treatment of Aβ also induced CEBPD activation in astrocytes [26]. The results of neurons growing in the CM from astrocyte expressing CEBPD or miR-135a provided a novel mechanism to explain the reduced neuronal neurite length and survival commonly observed in AD or other neuroinflammatory diseases. It also provided a new evidence for the communication between astrocytes and neuron in inflamed brain, especially in astrocyte-mediated cognitive loss. Moreover, AM135a, the antagomir neutralizing miR-135a, could enhance THBS1 levels to prevent neuronal loss and improve the learning ability in *AppTg* mice. The results indicated that administration of AM135a could be a therapeutically effective treatment for AD patients.

Previous studies have identified that several miRNAs in the brain are associated with AD, HD, PD, neuroviral infections, and schizophrenia [44, 45]. Upregulation of miR-135a was detected in the cerebrospinal fluid (CSF) of AD patients and a microarray profile of the brain of AD mice [46, 47]. miR-135a is a novel CEBPD responsive gene and first identified to regulate *THBS1* transcription. The results showed the first evidence of miRNA-dependent dysregulation of *THBS1* gene involved in the pathogenesis of AD. Consistent with previous studies [46, 47], our in situ hybridization result also demonstrated that miR-135a was increased in the cortex and hippocampus of *AppTg* mice. Whereas, a recent study showed that miR-135a targeted to beta-site amyloid precursor protein-cleaving enzyme 1 (BACE1) in neuron cells and downregulated in CSF and serum of *AppTg* mice [48]. The inconsistent results indicated that the regulation of miR-135a could be in a cell-type specific manner or because the variations of specimens of AD patients and age of the *AppTg* mice. In addition, BACE1 is majorly expressed and function in neuron cells. However, miR-135a is a glia-enriched miRNA and low in normal neuron cells [49]. Therefore, the contribution and impact of miR-135a-mediated BACE1 repression in AD patients need to be further verified.

In this study, we provided a result of CEBPD-regulated miRNAs in astrocytes (Table 2). In addition to miR-135a, several CEBPD-downregulated miRNAs including miR-9, miR-19b, miR-29a/b-1, miR-16, and miR-107 were found to be repressed in neurodegenerative diseases [50, 51]. Among them, the expression level of miR-9, miR-29a/b-1, and miR-107 was also reported to be negatively correlated with BACE1 in AD pathogenesis [52–54]. The attenuation of miR-9 and miR-29a were further found in HD mice, which resulted in the increase of transcription factor REST, a repressor of neurotrophic factor *BDNF* transcription [31]. In addition, with respect to PD, miR-19b, miR-29a, and miR-16 were decreased in sporadic and familial late-onset PD [55]. However, the specific location and regulation of above miRNAs in brain and their specific function in astrocytes still pose an open question for a further investigation. Furthermore, the links between CEBPD responsive miRNAs and putative downstream genes (Table 2) also provide interesting candidates for inhibiting release of neurotrophic factors or producing neurotoxins. Artemin (ARTN) is a potent neurotrophic factor for the peripheral nervous system and has great potential for the treatment of neuropathic pain [56]. We found that miR-1914 could be induced by CEBPD and is predicted to target the *ARTN* 3′-UTR region. In addition, neurotoxic factor nitric oxide generated via iNOS was found in postmortem brains of AD and PD patients [57]. CEBPD reduced the levels of miR-26b, miR-29a, and miR-128 that are predicted to target inducible nitric oxide synthase [10]. Therefore, the dissection of astrocytic CEBPD involvement in inflamed brains that could reveal more details for puzzling the complex issues in neurodegenerative disorders.

## Electronic Supplementary Material

Below is the link to the electronic supplementary material.

Fig. S1 Attenuated *Cebpd* expression in primary astrocytes does not inhibit neuronal viability. The conditioned medium came from either IL-1β-pretreated primary WT or *Cebpd* ^−/−^ astrocytes. Primary cortical neurons in WT and *Cebpd* ^−/−^ mice were grown with the mixture medium (9:1) of neuronal maintenance medium and above conditioned medium for 72 h. The MTT assay was conducted as indicated. Data are expressed as mean ± SEM by a Student’s *t* test. *P < 0.05, *NS* not significant. (GIF 19 kb)

High resolution image (TIFF 817 kb)

Fig. S2 CEBPD induces GLYCTK-AS1-001 expression. qRT-PCR confirmed that GLYCTK-AS1-001 levels from stable U373MG cells with zinc-inducible CEBPD expression system and then incubated in the presence or absence of 100 μM ZnSO_4_ for 6 h. The data represented the mean ± standard error of three independent experiments, each performed in triplicate. (*P < 0.05, Student’s *t* test). (GIF 9 kb)

High resolution image (TIFF 661 kb)

Fig. S3 Astrocytic CEBPD and miR-135a reduces neuronal nurite length. Conditioned medium (CM) from stable U373MG cells with zinc-inducible CEBPD (**a**) or with DOX-inducible miR-135a (**b**) expression system were subjected to exam THBS1 by Western blot and added to primary neurons for 48 h. **c** Attenuated miR-135a in U373MG cells restore the neuronal nurite outgrowth. Conditioned medium from stable U373MG cells with IPTG-inducible A-135a expression system with or without IL-1β treatment were subjected to exam THBS1 by western blot and added to primary neurons for 48 h. Neurons were stained with mouse anti-MAP2 Abs for morphological examination. ImageJ software was used to quantification micrometers of neurite length/neuron. Quantification of relative of neurite length/neuron. (*P < 0.05, **P < 0.01, Student’s *t* test) (GIF 63 kb)

High resolution image (TIFF 1928 kb)

Fig. S4 The expression of miR-135a is increased in the brain tissues lysate of *App*Tg mice. miR-135a level in wild-type, *AppTg*, and *AppTg/Cebpd* ^−*/*−^ mice was analyzed by qRT-PCR (*n* = 2 per genotype). The data represented the mean ± standard error of three independent experiments, each performed in triplicate. (***P < 0.001, Student’s *t* test) (GIF 13 kb)

High resolution image (TIFF 713 kb)

Fig. S5 The activity of caspases-3 reduced in brain tissues lysates of AM135a treated AppTg mice. The brain tissue lysate were extracted from wild-type (WT) mice and *App*Tg mice treated with scramble or AM135a (*n* = 2 per group). The lysate of each group were mixed with Caspase-Glo 3/7 reagent in 1:1 ration then incubate at room temperature. After 30 min, the luminescence of each sample were measured. (**P < 0.01, Student’s *t* test) (GIF 12 kb)

High resolution image (TIFF 707 kb)

Fig. S6 Negative control of IHC analysis. The tissue sections stained without primary antibody and only with second antibody which is Alexa Fluor® 488 or Alexa Fluor® 568. *Scale bar* = 100 μm. (GIF 37 kb)

High resolution image (TIFF 1307 kb)

ESM 1 (DOC 34.5 kb)
