# Supplementary figures and images for: Astrocytic CCAAT/Enhancer Binding Protein δ Regulates Neuronal Viability and Spatial Learning Ability via miR-135a
Source: Mol Neurobiol. 2015 Jul 26;53(6):4173–88. doi: 10.1007/s12035-015-9359-z (PMC4937099; doi:10.1007/s12035-015-9359-z)

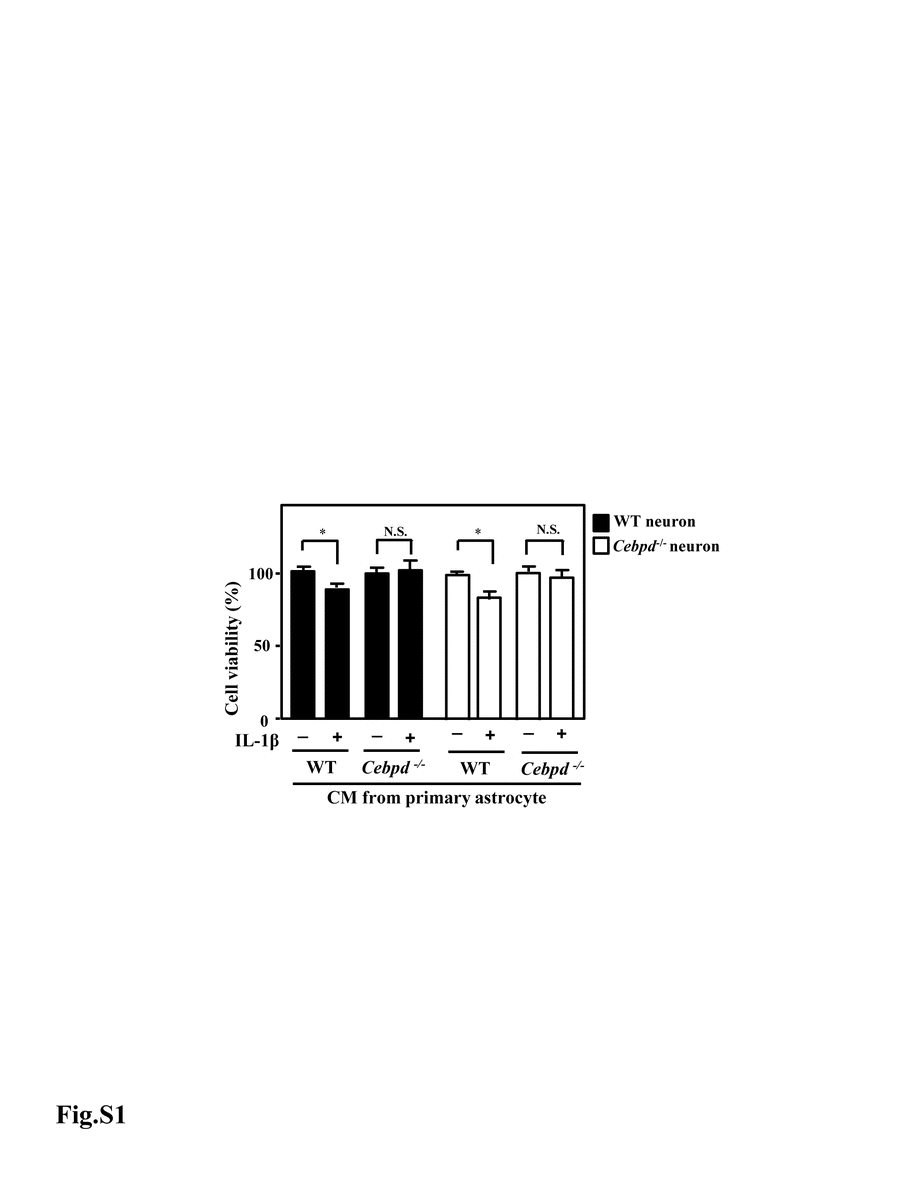

Supplement: Supplementary file 1 — Attenuated Cebpd expression in primary astrocytes does not inhibit neuronal viability. The conditioned medium came from either IL-1β-pretreated primary WT or Cebpd −/− astrocytes. Primary cortical neurons in WT and Cebpd −/− mice were grown with the mixture medium (9:1) of neuronal maintenance medium and above conditioned medium for 72 h. The MTT assay was conducted as indicated. Data are expressed as mean ± SEM by a Student’s t test. *P < 0.05, NS not significant. (GIF 19 kb) [file 12035_2015_9359_Fig7_ESM.gif]

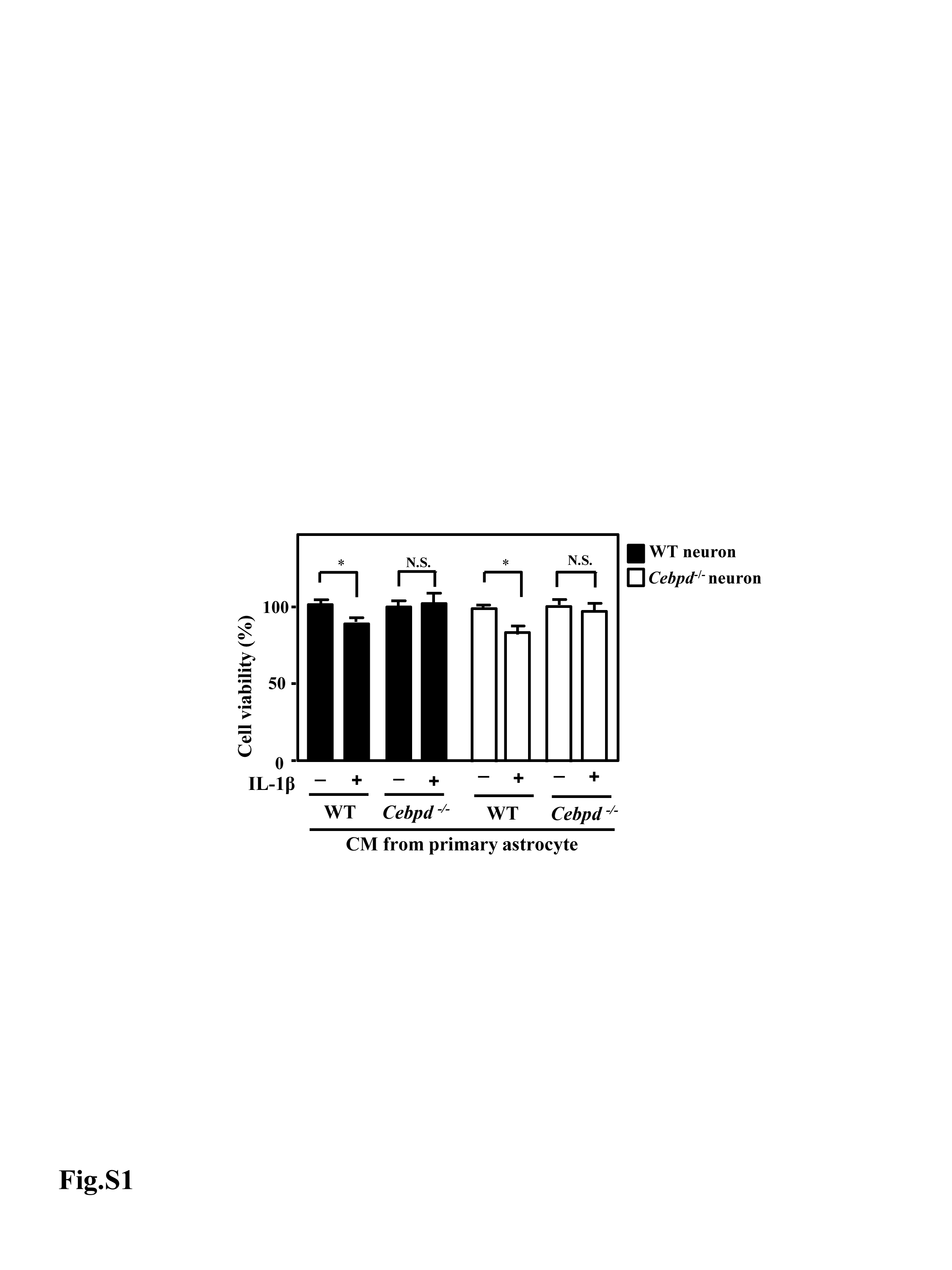

Supplement: Supplementary file 2 — High resolution image (TIFF 817 kb) [file 12035_2015_9359_MOESM1_ESM.tif]

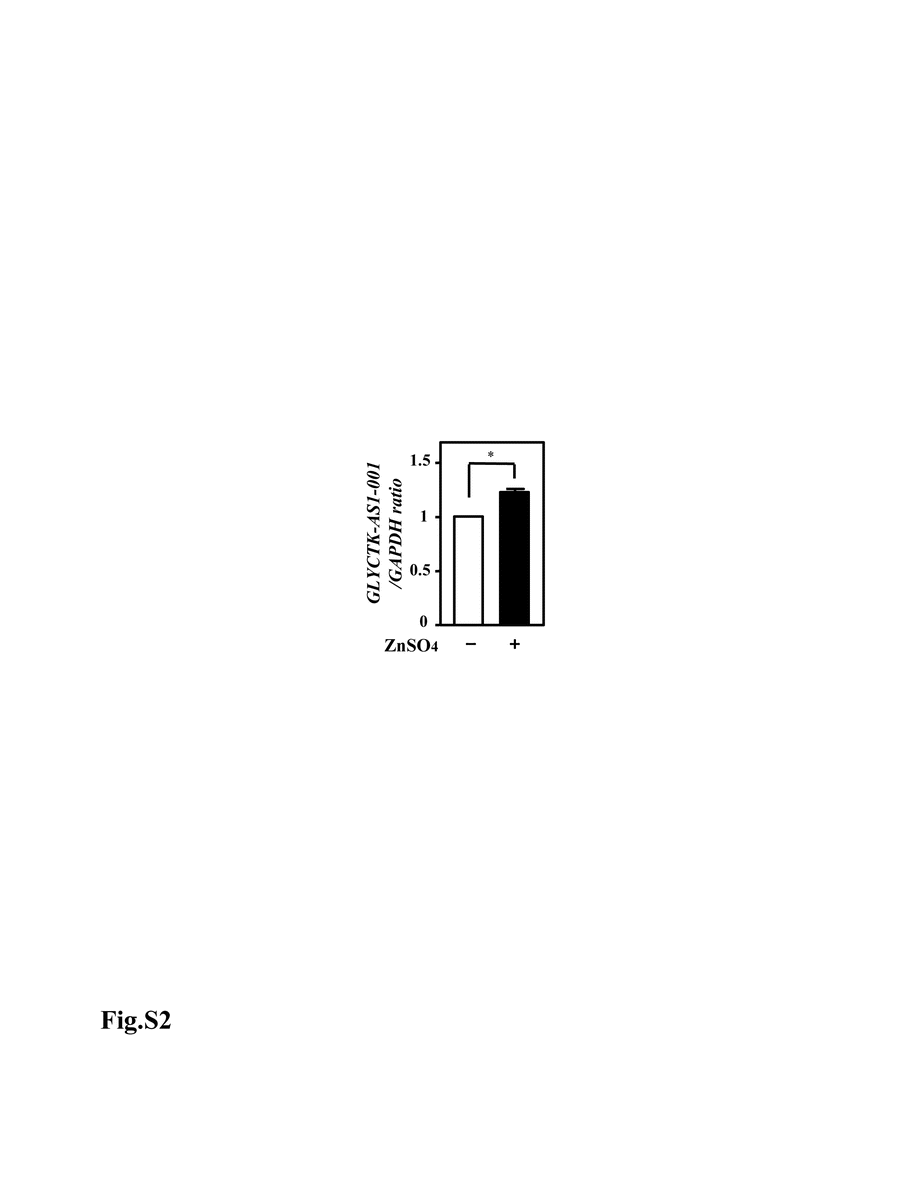

Supplement: Supplementary file 3 — CEBPD induces GLYCTK-AS1-001 expression. qRT-PCR confirmed that GLYCTK-AS1-001 levels from stable U373MG cells with zinc-inducible CEBPD expression system and then incubated in the presence or absence of 100 μM ZnSO4 for 6 h. The data represented the mean ± standard error of three independent experiments, each performed in triplicate. (*P < 0.05, Student’s t test). (GIF 9 kb) [file 12035_2015_9359_Fig8_ESM.gif]

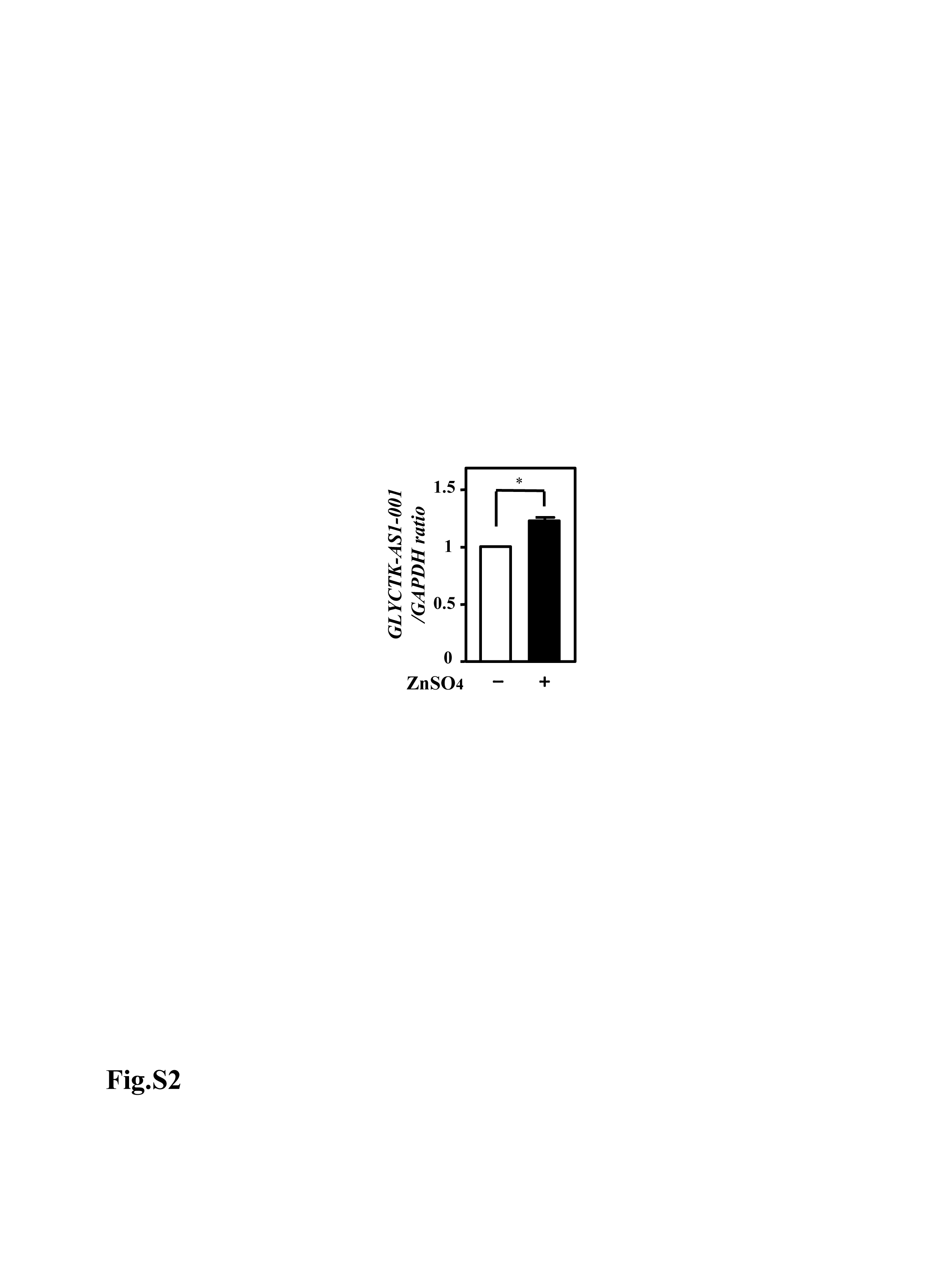

Supplement: Supplementary file 4 — High resolution image (TIFF 661 kb) [file 12035_2015_9359_MOESM2_ESM.tif]

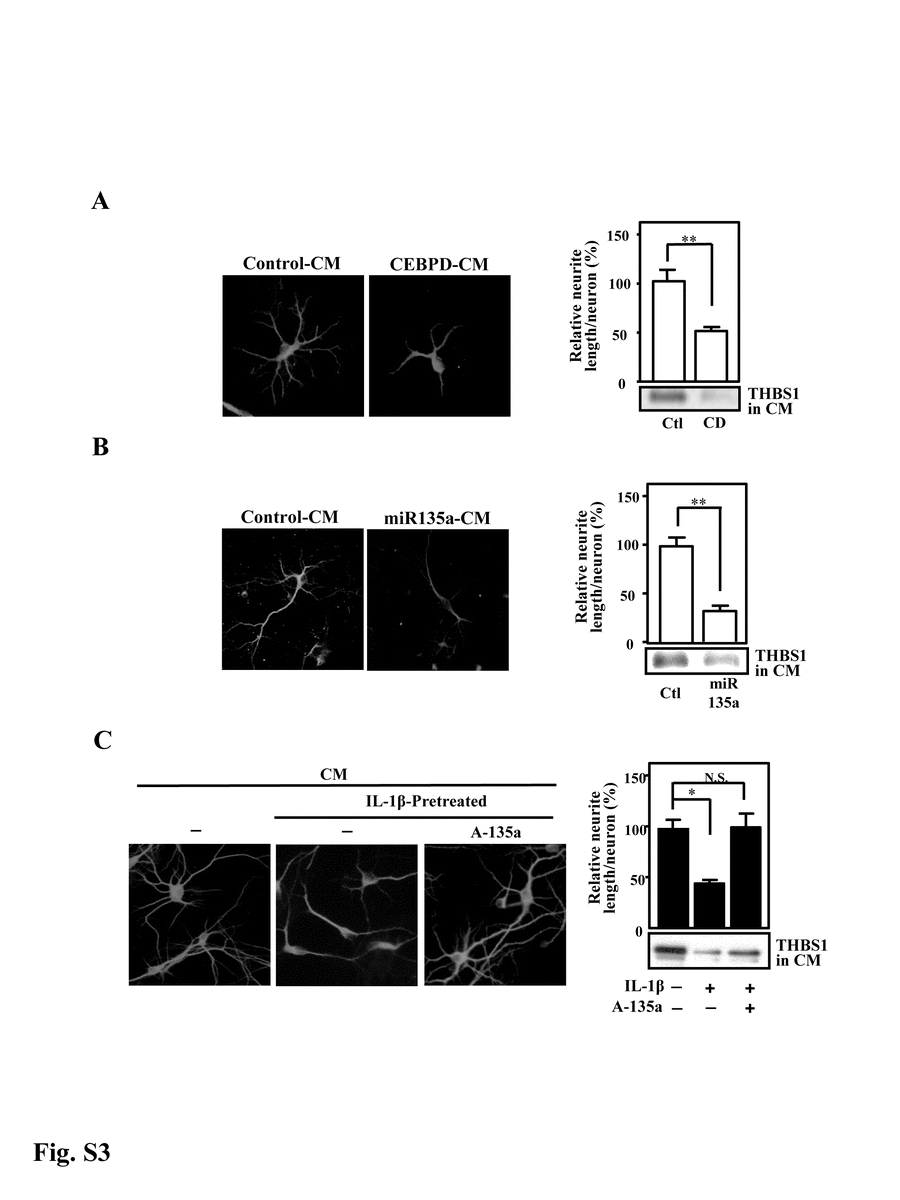

Supplement: Supplementary file 5 — Astrocytic CEBPD and miR-135a reduces neuronal nurite length. Conditioned medium (CM) from stable U373MG cells with zinc-inducible CEBPD (a) or with DOX-inducible miR-135a (b) expression system were subjected to exam THBS1 by Western blot and added to primary neurons for 48 h. c Attenuated miR-135a in U373MG cells restore the neuronal nurite outgrowth. Conditioned medium from stable U373MG cells with IPTG-inducible A-135a expression system with or without IL-1β treatment were subjected to exam THBS1 by western blot and added to primary neurons for 48 h. Neurons were stained with mouse anti-MAP2 Abs for morphological examination. ImageJ software was used to quantification micrometers of neurite length/neuron. Quantification of relative of neurite length/neuron. (*P < 0.05, **P < 0.01, Student’s t test) (GIF 63 kb) [file 12035_2015_9359_Fig9_ESM.gif]

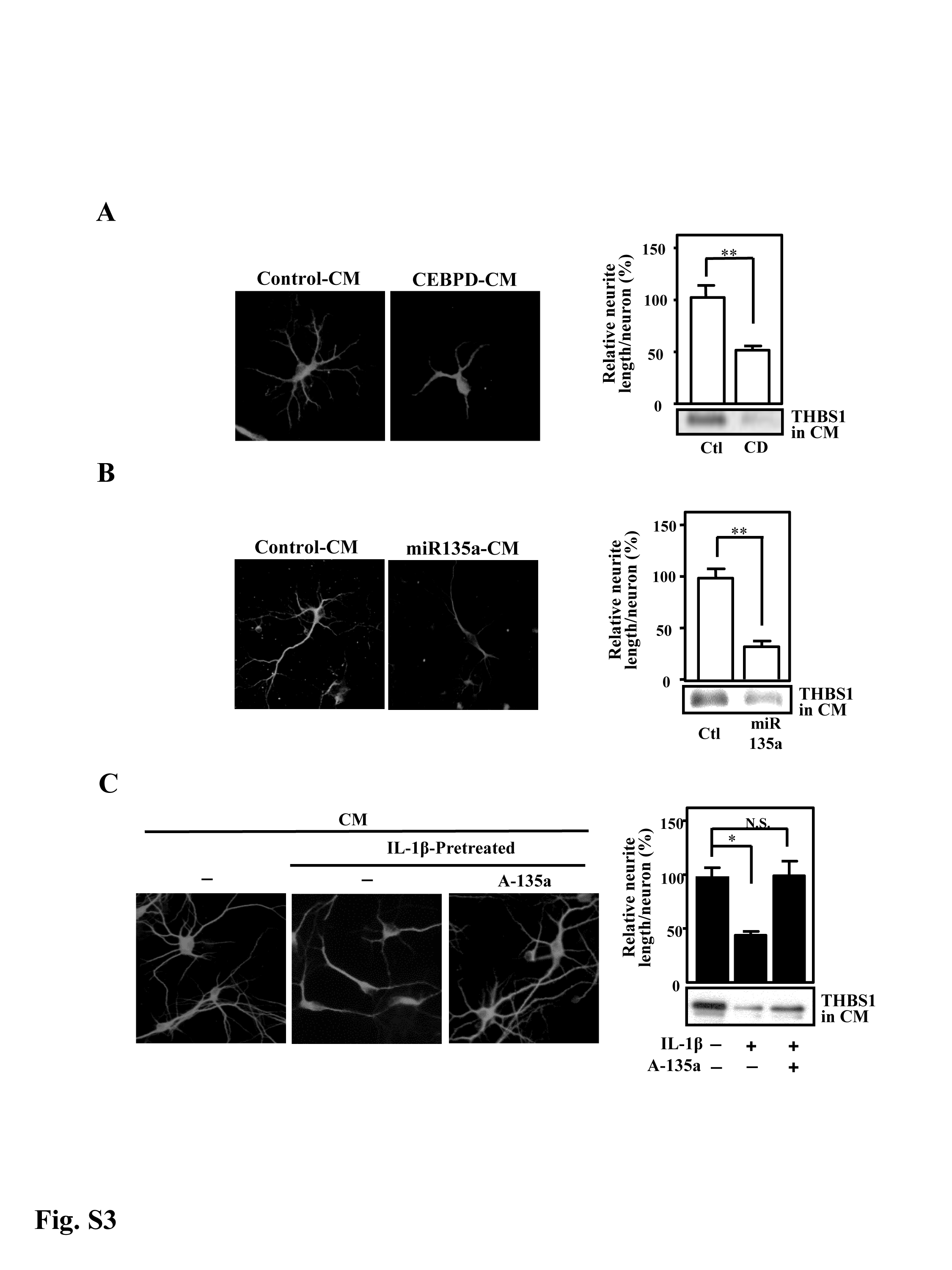

Supplement: Supplementary file 6 — High resolution image (TIFF 1928 kb) [file 12035_2015_9359_MOESM3_ESM.tif]

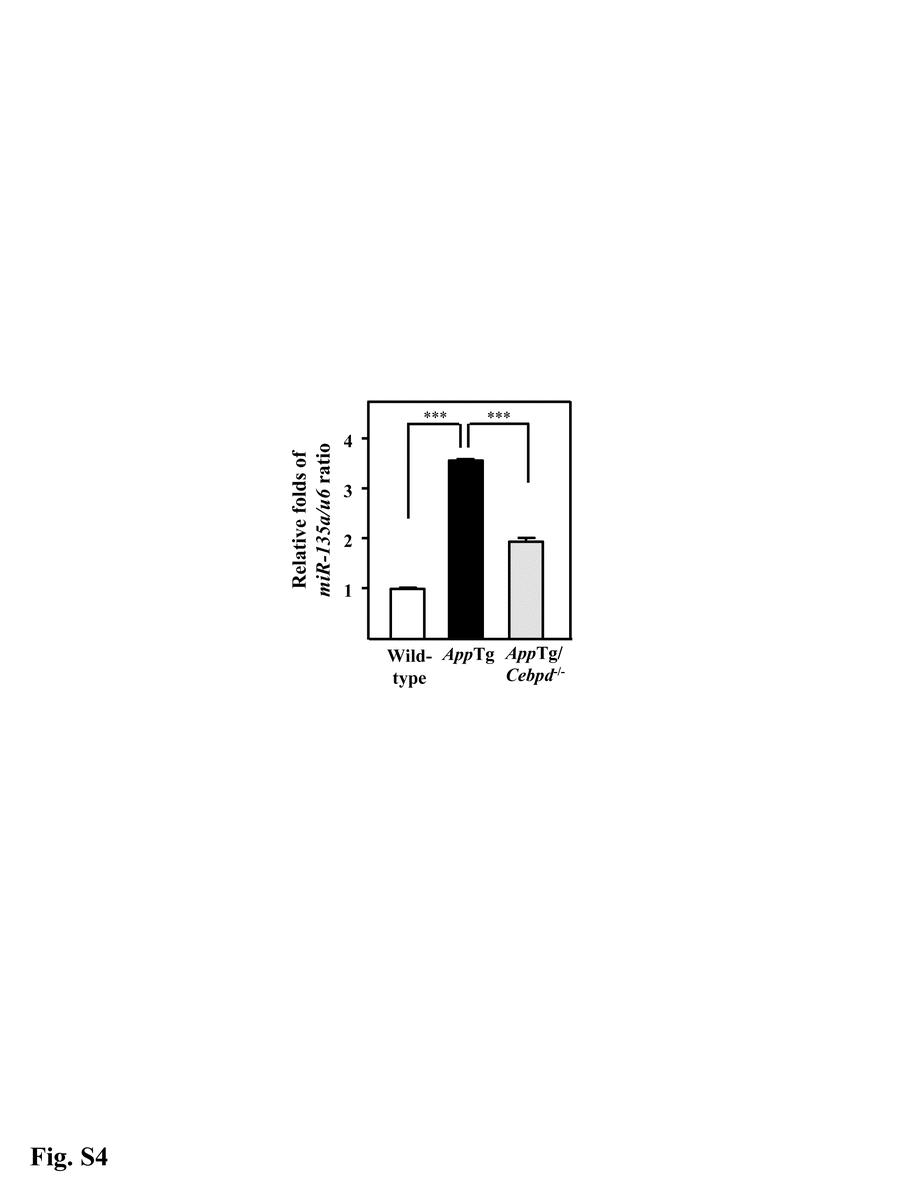

Supplement: Supplementary file 7 — The expression of miR-135a is increased in the brain tissues lysate of AppTg mice. miR-135a level in wild-type, AppTg, and AppTg/Cebpd −/− mice was analyzed by qRT-PCR (n = 2 per genotype). The data represented the mean ± standard error of three independent experiments, each performed in triplicate. (***P < 0.001, Student’s t test) (GIF 13 kb) [file 12035_2015_9359_Fig10_ESM.gif]

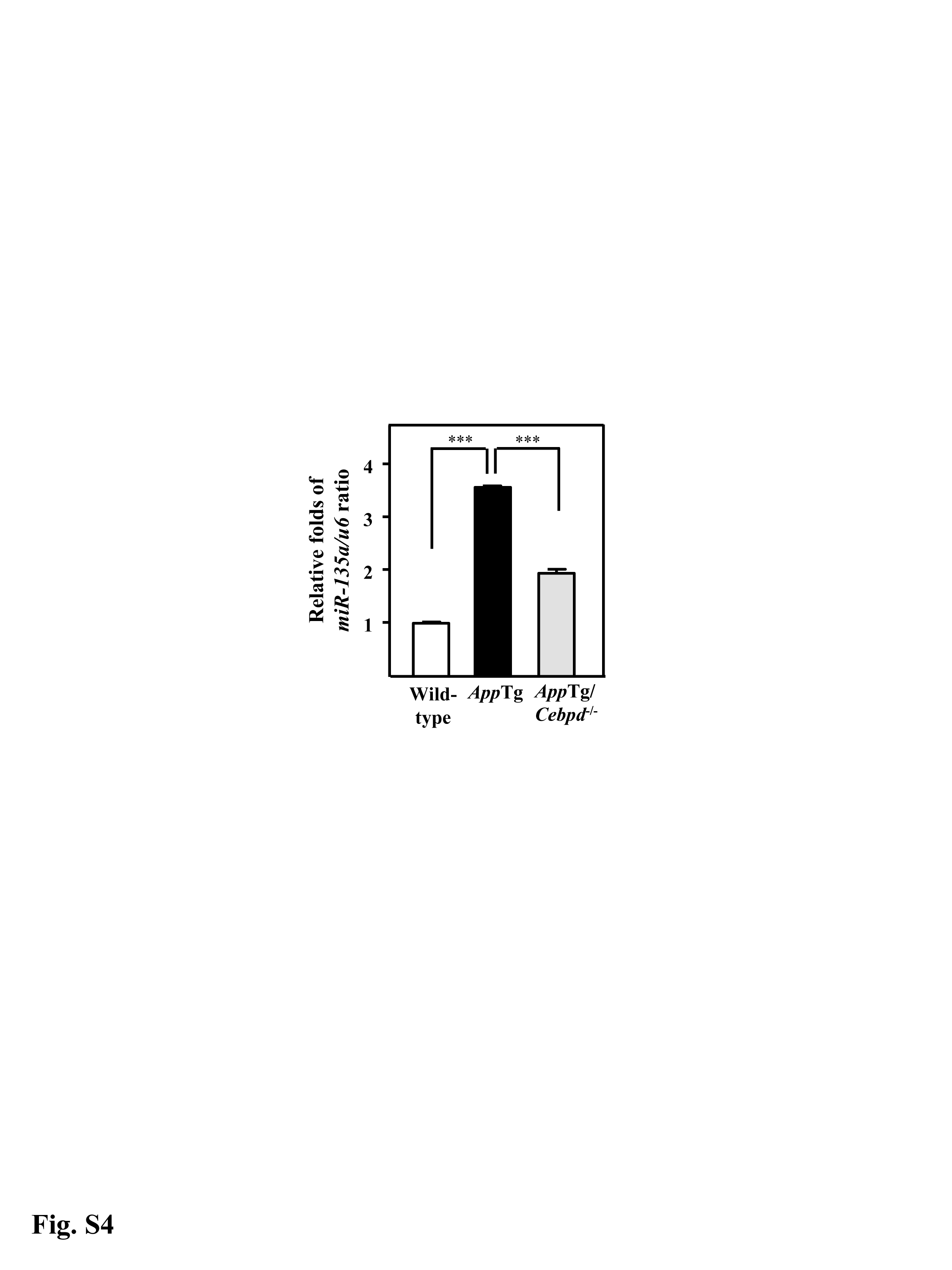

Supplement: Supplementary file 8 — High resolution image (TIFF 713 kb) [file 12035_2015_9359_MOESM4_ESM.tif]

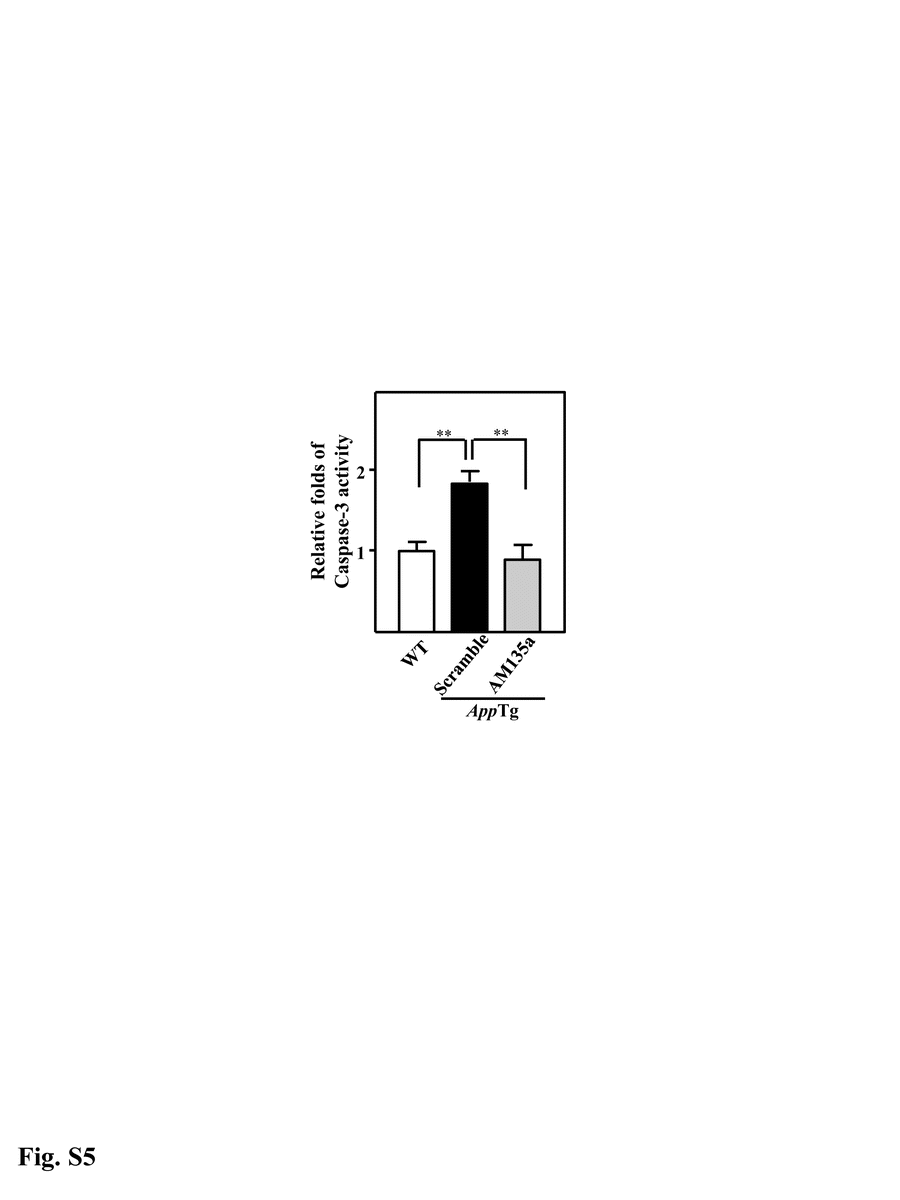

Supplement: Supplementary file 9 — The activity of caspases-3 reduced in brain tissues lysates of AM135a treated AppTg mice. The brain tissue lysate were extracted from wild-type (WT) mice and AppTg mice treated with scramble or AM135a (n = 2 per group). The lysate of each group were mixed with Caspase-Glo 3/7 reagent in 1:1 ration then incubate at room temperature. After 30 min, the luminescence of each sample were measured. (**P < 0.01, Student’s t test) (GIF 12 kb) [file 12035_2015_9359_Fig11_ESM.gif]

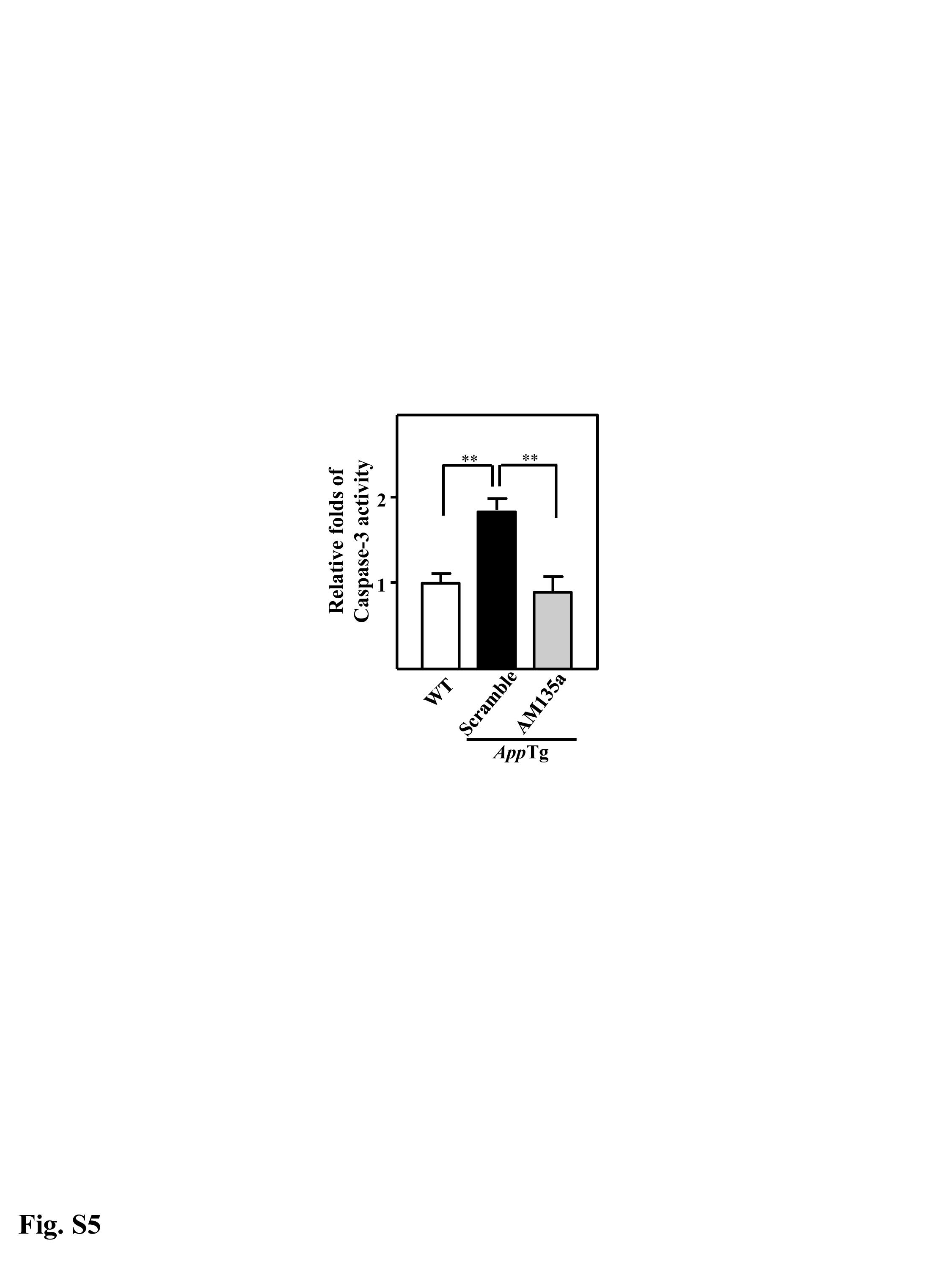

Supplement: Supplementary file 10 — High resolution image (TIFF 707 kb) [file 12035_2015_9359_MOESM5_ESM.tif]

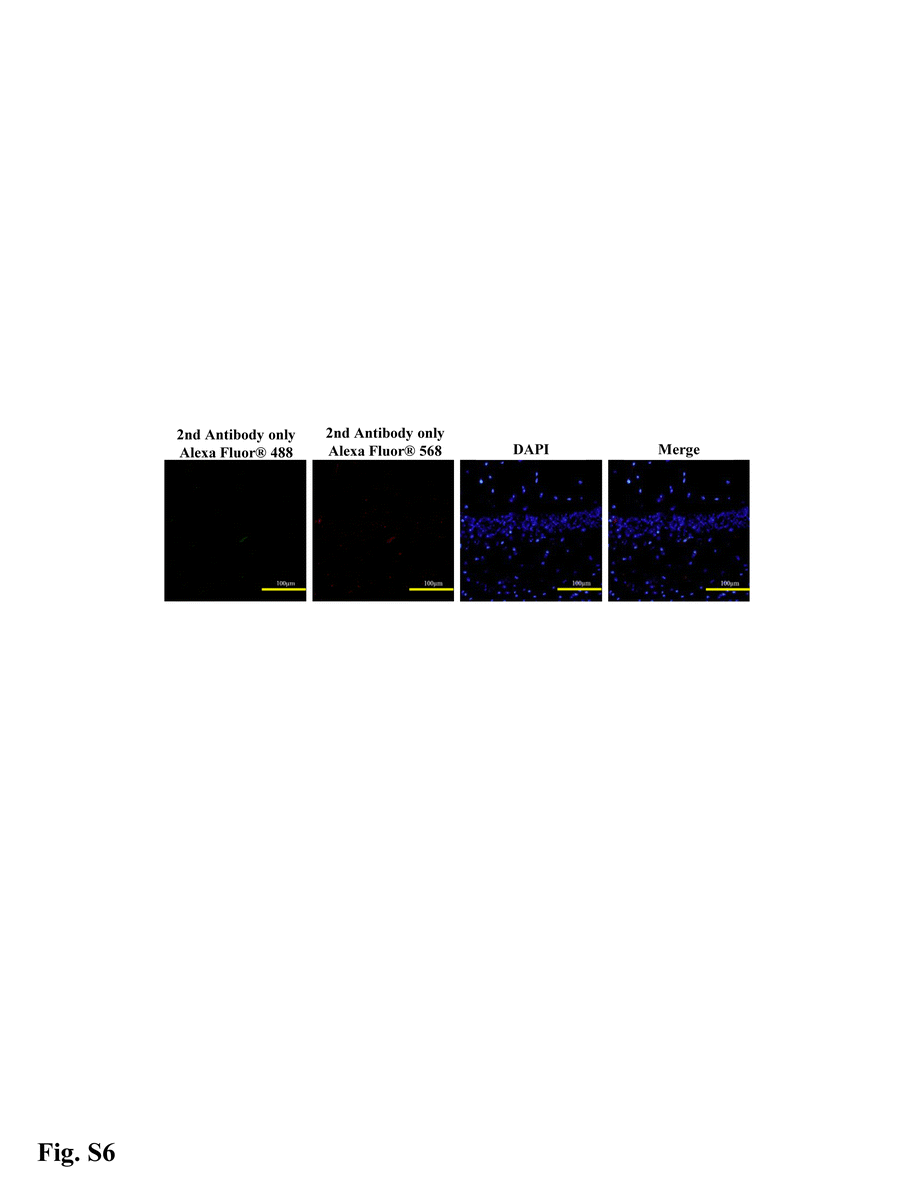

Supplement: Supplementary file 11 — Negative control of IHC analysis. The tissue sections stained without primary antibody and only with second antibody which is Alexa Fluor® 488 or Alexa Fluor® 568. Scale bar = 100 μm. (GIF 37 kb) [file 12035_2015_9359_Fig12_ESM.gif]

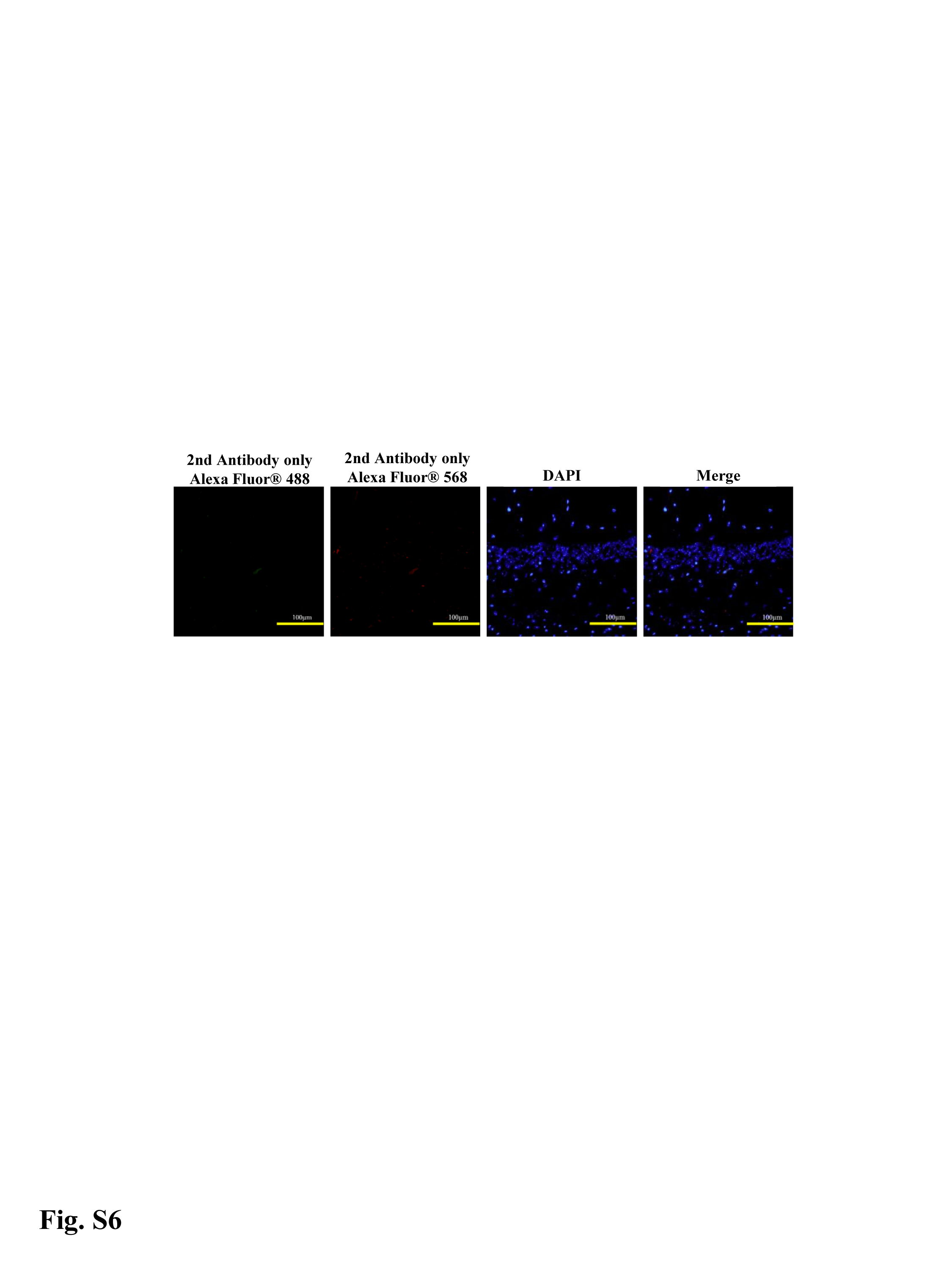

Supplement: Supplementary file 12 — High resolution image (TIFF 1307 kb) [file 12035_2015_9359_MOESM6_ESM.tif]
