# Supplementary material for: Astrocytic CCAAT/Enhancer Binding Protein δ Regulates Neuronal Viability and Spatial Learning Ability via miR-135a
Source: Mol Neurobiol. 2015 Jul 26;53(6):4173–88. doi: 10.1007/s12035-015-9359-z (PMC4937099; doi:10.1007/s12035-015-9359-z)
Supplement: Supplementary file 13 — (DOC 34.5 kb) [file 12035_2015_9359_MOESM7_ESM.doc]

**Supplementary Figure legends:**

**Fig. S1 Attenuated *Cebpd* expression in primary astrocytes does not inhibit neuronal viability.** The conditioned medium came from either IL-1β-pretreated primary WT or *Cebpd*^-/-^ astrocytes. Primary cortical neurons in WT and *Cebpd*^-/-^ mice were grown with the mixture medium (9:1) of neuronal maintenance medium and above conditioned medium for 72 h. The MTT assay was conducted as indicated. Data are expressed as mean ± SEM by a Student’s t test. *, P < 0.05; NS: not significant.

**Fig. S2** **CEBPD induces GLYCTK-AS1-001 expression.** qRT-PCR confirmed that GLYCTK-AS1-001 levels from stable U373MG cells with zinc-inducible CEBPD expression system and then incubated in the presence or absence of 100 μM ZnSO4 for 6 h. The data represented the mean ± standard error of 3 independent experiments, each performed in triplicate. (*p < 0.05, Student’s t-test).

**Fig. S3 Astrocytic CEBPD and miR-135a reduces neuronal nurite length.** Conditioned medium (CM) from stable U373MG cells with zinc-inducible CEBPD **(A)** or with DOX-inducible miR-135a **(B)** expression system were subjected to exam THBS1 by western blot and added to primary neurons for 48 hours. **(C)** Attenuated miR-135a in U373MG cells restore the neuronal nurite outgrowth. Conditioned medium from stable U373MG cells with IPTG-inducible A-135a expression system with or without IL-1β treatment were subjected to exam THBS1 by western blot and added to primary neurons for 48 hours. Neurons were stained with mouse anti-MAP2 Abs for morphological examination. ImageJ software was used to quantification micrometers of neurite length /neuron. Quantification of relative of neurite length/neuron. (*p < 0.05, **p < 0.01, Student’s t-test)

**Fig. S4 The expression of miR-135a is increased in the brain tissues lysate of *App*Tg mice.** miR-135a level in wild-type, *AppTg*, and *AppTg/Cebpd^-/-^* mice was analyzed by qRT-PCR (n=2 per genotype). The data represented the mean ± standard error of 3 independent experiments, each performed in triplicate. (***p < 0.001, Student’s t-test)

**Fig. S5 The activity of caspases-3 reduced in brain tissues lysates of AM135a treated AppTg mice.** The brain tissue lysate were extracted from wild-type (WT) mice and *App*Tg mice treated with scramble or AM135a (n=2 per group). The lysate of each group were mixed with Caspase-Glo 3/7 reagent in 1:1 ration then incubate at room temperature. After 30 minutes, the luminescence of each sample were measured. (**p < 0.01, Student’s t-test)

**Fig. S6 Negative control of IHC analysis.** The tissue sections stained without primary antibody and only with second antibody which is Alexa Fluor® 488 or Alexa Fluor® 568. Scale bar = 100 µm.
